# A comprehensive analysis of the *Lactuca sativa,* L. transcriptome during different stages of the compatible interaction with *Rhizoctonia solani*

**DOI:** 10.1038/s41598-019-43706-5

**Published:** 2019-05-10

**Authors:** Bart Verwaaijen, Daniel Wibberg, Anika Winkler, Rita Zrenner, Hanna Bednarz, Karsten Niehaus, Rita Grosch, Alfred Pühler, Andreas Schlüter

**Affiliations:** 1 Center for Biotechnology, Bielefeld University, Bielefeld, Germany; 2 Leibniz-Institute of Vegetable and Ornamental Crops (IGZ), Großbeeren, Germany; 3 Present Address: Computational Biology, Faculty of Biology, Bielefeld University, Bielefeld, Germany

**Keywords:** Plant immunity, Biotic

## Abstract

The leafy green vegetable *Lactuca sativa*, L. is susceptible to the soil-born fungus *Rhizoctonia solani* AG1-IB. In a previous study, we reported on the transcriptional response of *R. solani* AG1-IB (isolate 7/3/14) during the interspecies interaction with *L. sativa* cv. Tizian by means of RNA sequencing. Here we present the *L. sativa* transcriptome and metabolome from the same experimental approach. Three distinct interaction zones were sampled and compared to a blank (non-inoculated) sample: symptomless zone 1, zone 2 showing light brown discoloration, and a dark brown zone 3 characterized by necrotic lesions. Throughout the interaction, we observed a massive reprogramming of the *L. sativa* transcriptome, with 9231 unique genes matching the threshold criteria for differential expression. The lettuce transcriptome of the light brown zone 2 presents the most dissimilar profile compared to the uninoculated zone 4, marking the main stage of interaction. Transcripts putatively encoding several essential proteins that are involved in maintaining jasmonic acid and auxin homeostasis were found to be negatively regulated. These and other indicator transcripts mark a potentially inadequate defence response, leading to a compatible interaction. KEGG pathway mapping and GC-MS metabolome data revealed large changes in amino acid, lignin and hemicellulose related pathways and related metabolites.

## Introduction

The leafy green vegetable lettuce (*Lactuca sativa*, L.), member of the family *Asteraceae*, is popular around the world for food and feed purposes. Unfortunately, lettuce is sensitive to a wide range of bacterial, viral and fungal pathogens. The necrotrophic soil-borne pathogen *Rhizoctonia solani* AG1-IB is best known for bottom rot disease on lettuce^1^.

The inherent plant defence mechanism reactions are specific for pathogens with different lifestyles like necrotrophs, biotrophs and hemibiotrophs^2^. Obligate biotrophic pathogens (e.g., *Puccinia* spp., *Blumeria* spp.) have coevolved mechanisms to maintain host viability, use nutrients from living cells and generally lack toxin production^3^. Hemibiotrophic pathogens such as *Phytophthora infestans* are characterized by their capability to switch from a biotrophic lifestyle to a necrotrophic lifestyle^4^. In contrast to biotrophs, necrotrophs exclusively extract nutrients from dead cells and have evolved aggressive and extensive virulence strategies that promote host cell death^5^. Necrotrophs include host-specific like *Cochliobolus carbonum* and broad host-range species such as *Botrytis cinerea*, *Sclerotinia sclerotiorum*, and *Alternaria brassicicola*^5^. The soil-borne pathogen *R. solani* is generally considered to be a simple necrotrophic broad-host-range pathogen^6^.

Regardless of the pathogens lifestyle, the activation of plant defence responses involves complex events on cellular, histological and molecular levels. The response of plants against pathogens generally operates at two levels. The first level is induced in plants when microbe associated molecular patterns (MAMPs) or damage associated molecular patterns (DAMPS) are recognized by pattern recognition receptors (PRR) which trigger a broad-spectrum defence response known as pattern-triggered immunity (PTI). The second level, the effector-triggered immune response (ETI), is induced through recognition of pathogen specific avirulence effectors by host-disease resistance proteins that result in rapid and robust response^7^. The ETI response is based on the principle of gene-for-gene resistance. Against necrotrophic fungal pathogens, this mode of defence is rather rare in plants^8^ and not known for *R. solani* interaction in any host plant to date. Plant defence responses to necrotophs are very complex and reflect the multiplicity of virulence mechanisms of necrotrophs targeting diverse cellular processes^8^. As already mentioned, infections of necrotrophic pathogens result in host cell death. To induce host cell necrosis and leakage of nutrients, necrotrophs can secrete phytotoxic secondary metabolites and peptides, cell wall-degrading enzymes and produce reactive oxygen species^4^. Plant host cell death in turn is associated for instance with production of antimicrobial peptides and hormones such as salicylic acid (SA), ethylene (ET), and jasmonic acid (JA), accumulation of reactive oxygen species (ROS) or modification of cell wall components^5^. The role of host cell death differs between plants responding to biotrophic or necrotrophic pathogens. Cell death (hypersensitive response) induced at the site of infection by biotrophs is an indicator of resistance, whereas host cell death induced by necrotrophs is an indicator for successful infection. Therefore, based on the pathogen’s lifestyle, different responses are required from the plant to launch an effective defence. The response against biotrophs mostly relies on SA, whereas the response against necrotrophs predominantly depends on JA and ET regulated pathways. However, nowadays it is known that separation between these mechanisms is not the schism that it was formerly presumed to be. Several studies have pointed at the existence of interplay between these mechanisms of plant defence^9^.

In addition, it has been suggested that several necrotrophic pathogens are capable of tricking the plant defence system by activating the response towards biotrophs, resulting in host cell death and thus promoting a successful interaction of the necrotrophic pathogen^10^. Little is known on the plant’s control of disease-associated cell death especially as a response to interaction with a broad host-range necrotrophic pathogen such as *R. solani*. Besides lettuce, the necrotrophic pathogen *R. solani* AG1-IB affects many more host plants^11^. First insights into the *R. solani* AG1-IB transcriptome during interaction with lettuce in a leaf infection model showed high expression of *R. solani* agglutinin genes as well as genes associated with apoptosis. Moreover, 21 genes of unknown function were specifically expressed in the initial phase of interaction, symptomless zone 1^12^. These findings suggest that the early phase of *R. solani* AG1-IB interaction is associated with mechanisms leading to host cell death. However, little is known about the biochemical mechanisms specifying this pathosystem. To elucidate the various interactions involved, we previously reported on the genome sequence of lettuce cv. Tizian and *R. solani* AG1-IB (isolate 7/3/14), addressing the genomic potential and transcriptional activity of both partners in the course of their interaction^13–16^. *R. solani* harbours various genes putatively specifying functions that can be linked to pathogenicity like: MAP kinases, GABA metabolism, melanin synthesis, plant cell wall degradation and interference with plant cell defences^13^. Concerning the lettuce cv. Tizian genome, we previously identified a high number of genes associated with plant defences^17^. Moreover, substantial research efforts have been conducted for the lettuce cv. Salinas^18–22^. Previously, the structure of the major resistance cluster and the identification of pathogen-recognizing receptors were reported for this cultivar^23,24^. First insights into the response of lettuce towards the necrotrophic fungus *Botrytis cinerea* uncovered upregulation of the phenylpropanoid and terpenoid synthesis pathways as well as a global downregulation of photosynthesis^22^.

To date our understanding of plant defence mechanisms, especially against attack by necrotrophic pathogens such as *R. solani,* is limited and more knowledge of the plant defence against these pathogens is needed for future control strategies. After the detailed analysis of the *R. solani* AG1-IB transcriptome during interaction with lettuce in a previous publication^12^. We here investigate lettuce cv. Tizian during the interaction with *R. solani* AG1-IB by means of transcriptome and metabolome analysis within a detached leaf model (DLM)^25–27^.

## Methods

### Experimental setup

**Figure 1:**
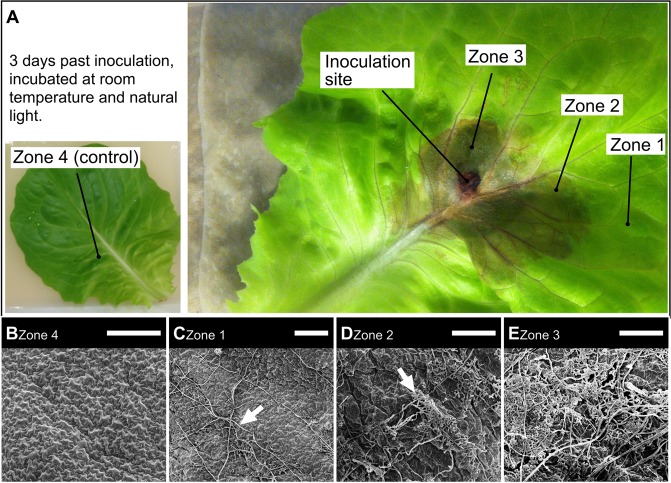
(**A**) Exemplary representation of the previously published lettuce leaf interaction model that was used in the current study^12^. (**B**–**E**) Scanning electron microscopy images, ruler size equals 200 μm. (**B**) Zone 4 represents the blank (non-inoculated lettuce leaf). (**C**) Zone 1 is most distal from the inoculation site and symptomless; no differentiated infection structures were observed in this zone. Exemplary runner hyphae are indicated by an arrow. (**D**) Zone 2 represents the intermediate zone where both living lettuce tissue as well as *R. solani* AG1-IB infection structures were visible, exemplary infection structures are indicated by an arrow. (**E**) Zone 3 is the macerated zone directly around the inoculation site.

The experimental design, cultivation of lettuce (cv. Tizian) and the pathogen *R. solani* AG1-IB (isolate 7/3/14), RNA extraction and the RNAseq sequencing on the Illumina Hiseq platform was done as described by Verwaaijen *et al*.^12^. Briefly, detached leafs from *L. sativa* cv. Tizian were inoculated with *R. solani* AG1-IB isolate 7/3/14 (Fig. 1) and incubated for 3 days at room temperature. After incubation with the pathogen, RNA was extracted from three distinct interaction zones with increasing extend (degree) of interaction (zone 1, 2, and 3 as illustrated and defined in Fig. 1) and a fourth sample from non-inoculated leafs (zone 4). Three plants were used to create biological replicates, with eight leafs per plant to obtain sufficient material.

### Mapping of RNA-Seq reads

The sequencing data from EBI ArrayExpress accession E-MTAB-4762 were mapped using Tophat 2 (v2.1.0) onto the *R. solani* AG1-IB 7/3/14 genome (EMBL LN679100–LN679996)^28^. Subsequently, the unmapped reads were converted back to FASTQ format (SAMtools) and mapped (Tophat 2) onto the lettuce cv. Tizian genome (CoGe genome ID:36441). The double mapping approach was used to minimize interference from sequencing artefacts and homologous regions between both species that may influence the analysis of transcriptional levels.

### Transcription analysis

Sequence reads were counted in the ReadXplorer platform 2.0.1^29,30^. Reads per kilo-base per million reads (RPKM) values were extracted for principle component analysis (PCA) and to determine the most abundant transcripts per sample. PCA plots based on RPKM values were calculated and visualized with ClustVis for Docker^31,32^. The DESeq^33^ R package was used as implemented for differential gene expression analysis, counting only uniquely mapped reads and using the zone 4 (blank; non-inoculated lettuce leaf tissue) as reference. DEGs (differentially expressed genes) featuring fold-changes larger or smaller than 2 or −2 with corresponding p-adjusted values below 0.05 were deemed significant for all further analyses unless defined differently^34^. In addition, Upset was used to depict the distribution of differentially expressed genes between the pairwise DESeq comparisons^35^. Differentially expressed resistance gene (R-gene) candidates were depicted based on RPKM values as heat map through the ClustVis platform^31^.

### Functional enrichment of expressed genes

Translated proteins derived from the non-redundant transcriptome based on the *L. sativa* gene prediction were compared with the *Arabidopsis thaliana* proteins from UniProt to retrieve orthologous candidates (Blast cut-off expectancy value 1 × 10^−11^). These proteins were ordered according to the fold-changes of the respective DESeq outputs and uploaded into the GOrilla platform, for enrichment of gene ontology terms (GO)^36^. All fold-change data were included; no filtering was applied. For a second GO-based approach, GO terms were counted from the transcript groups previously generated with Upset^35^ and compared to the GO term counts for the complete genome. Statistical validity of enrichment values was checked with a single tailed Fisher’s exact test at a 0.05 significance limit. Only Upset groups with 100 DEGs or more were analysed.

The Mercator pipeline was used at default settings to provide a functional annotation mapping file for MapMan enrichment analysis^37,38^. The DESeq fold-changes were imported in the MapMan platform for visualization and enrichment of functional bins. Enrichment of the bins was tested with the Wilcoxon rank sum test, corrected for false discovery rate (FDR).

Kyoto Encyclopedia of Genes and Genomes (KEGG)^39–41^ Ontology (KO) terms were linked to the differentially expressed genes to obtain pathway mappings using the KEGG Automatic Annotation Server (KAAS)^42^. Visualizations were generated with the KEGG mapper tool and custom heat map scaling created in Microsoft Excel.

### Scanning Electron Microscopy

Infected plant samples were fixed in 2% para-formaldehyde in phosphate buffer (100 mM, pH 7.2) for two hours on ice. The samples were washed three times for 20 minutes each in the same buffer. The samples were further fixed in 2% Osmium tetroxide for one hour and thereafter carefully washed to remove all fixative. Dehydration was carried out in a series of acetone starting at 20% going up to 100% in 10% steps. 100% acetone was replaced by 100% acetone that was stored on a molecular sieve (4 Å) to remove remaining traces of water. The samples were transferred to a critical point dryer (Balzers CPD 030, Switzerland). Carbon dioxide (water free, Linde, Germany) was used as transitional fluid at 73.8 bar and 31 °C. Dry samples were mounted on SEM-sample holders (Plano, Germany) and sputtered with 10 nm Gold using a Balzers SCD004 sputter. Samples were analyzed sing a S-450 scanning microscope (Hitachi; Japan) at 15 kV. Images were recorded using a DISS5 acquisition system (Point Electronics, Germany).

### Metabolome analysis

#### Metabolite extraction & Gas chromatography – mass spectrometry

Detached leafs from *L. sativa* cv. Tizian were inoculated with *R. solani* AG1-IB isolate 7/3/14 and the different interaction zones were dissected, as was described in Verwaaijen *et al*. 2017^12^. The plant material was frozen in liquid nitrogen and lyophilized. The dry plant material was homogenized to a uniform powder using a laboratory blender (IKA, Staufen, Germany). Approximately 8 mg of the samples were homogenized and extracted using a Ribolyzer (Bertin, Frankfurt Main, Germany) at 3 × 45 sec, 6.5 m/s with 0.5 g of silica beads and 1 ml 80% methanol containing 10 µM ribitol as internal standard. The dry weight of all samples was determined and documented for later normalization.

After centrifugation, 750 µl of the supernatant was dried in a stream of nitrogen gas and transferred to the MultiPurpose Sampler (MPS2, Gerstel, Germany). There the samples have been subsequently derivatized with 50 µl Methoxylamin-hydrochloride (20 mg/ml in pyridine, Sigma Aldrich) and 50 µl MSTFA (Macherey-Nagel), as well as injected for the analysis in Leco Pegasus IV gas chromatograph coupled to time-of-flight mass spectrometer (Leco, Saint Joseph, USA). The GC-instrument was equipped with a Optima^®^-5MS Accent column (30 m, ID 0.25, df 0.25 µm; Macherey-Nagel). 1 µl sample was injected (splitless) for GC-MS analysis. The oven program was: 3 minutes 80 °C, ramp with 5 °C per minute up to 325 °C, isothermic conditions for 2 minutes at 325 °C. Transfer line temperature was set on 250 °C and ion source on 220 °C. Mass spectra were recorded from m/z 50 to 750.

#### Data analysis with the Xcalibur

The resulted chromatogram and mass spectra were converted to Thermo RAW data format. Data analysis as peak picking, database search and integration of peak areas of selected extracted ion chromatograms (EICs) was carried out by the Xcalibur 2.0.7 software (Thermo Fisher Scientific, USA). Peak identification was carried out by comparison of retention times and mass spectra of reference compounds that were analyzed under the same conditions. In addition, a comparison of all mass spectra information with the Golm Metabolite Database (GMD) and the database of the National Institute of Standards and Technology (NIST) was performed. The confidence level was assigned to every metabolite identification in accordance to the proposed reporting standards of the Chemical Analysis Working Group^43^ ranging from (1): identified by the measurement of the chemical reference standard, (2): putatively identified by significant database hit, (3): putatively characterized by database hit as compound of a certain chemical class, (4): unknown compound.

The software approach delivers an Excel Table containing the identified metabolites and their quantification normalized to the dry weight and ribitol, as well as a ANOVA analysis for significance. Visual presentation of PCA and heatmaps were generated with ClustVis^31^.

## Results

### Lettuce/*Rhizoctonia solani* leaf interaction model

Previously, we presented results of *R. solani* AG1-IB transcriptomes using the lettuce leaf model^12^. In this study, we specifically focus on the lettuce transcriptome in the course of interaction. The experiment was conducted as follows. Lettuce cv. Tizian leafs were inoculated with *R. solani* AG1-IB isolate 7/3/14 and incubated for 3 days (Supplementary Video 1). During incubation, three distinct zones were observed on the inoculated leafs. These zones were microscopically investigated and subsequently sampled for RNA extraction and sequencing (Fig. 1).

### Differential expression of lettuce genes during interaction with *R. solani*

**Figure 2:**
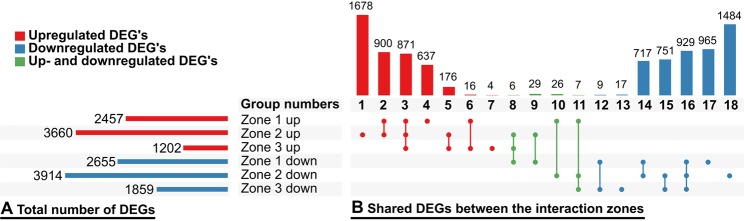
Significantly differentially expressed genes (DEGs) among the depicted pairwise comparisons as generated with DESeq using zone 4 (blank) as reference. Only transcripts with a p-adj values of 0.05 or less and an absolute fold-change of 2-fold or more have been plotted. (**B**) The horizontal bar chart represents the total amount of significant DEGs in each comparison distinguished between up- and down-regulated transcripts. (**C**) The vertical bar chart depicts the amount of shared transcripts between the groups that are defined by the connected dots matrix in the form of an Upset plot^35^. The DESeq calculations and Upset groups can be found in Supplementary Table S2 and 3.

In order to quantify the transcriptional response of lettuce to *R. solani* AG1-IB attack at different phases of interaction, differential transcriptome analyses of the interaction zones were carried out with DESeq, between the zone 4 sample and the three interaction zones. These results were merged in an Upset plot to investigate the distribution of significantly differentially expressed genes (DEGs) between the different comparisons (Fig. 2). Considering all three zones of interaction, 15,747 events of significant differential expression were detected with a p-adj value of 0.05 or less and an absolute fold-change of 2-fold or more. 9,231 of these DEGs correspond to unique transcripts representing about 50% of all non-redundant transcripts. Respectively, 4,282 DEGs were upregulated and 4,872 were downregulated. It is noteworthy that zone 2 features the highest number of uniquely up- as well as uniquely down-regulated transcripts. Zone 1 shows the second most uniquely transcribed transcripts. Zone 3, the most advanced interaction phase, has only few uniquely transcribed genes, so no or only little transcriptional changes occur here, as compared to zone 1 and 2.

### Biological functions of lettuce DEGs

**Figure 3:**
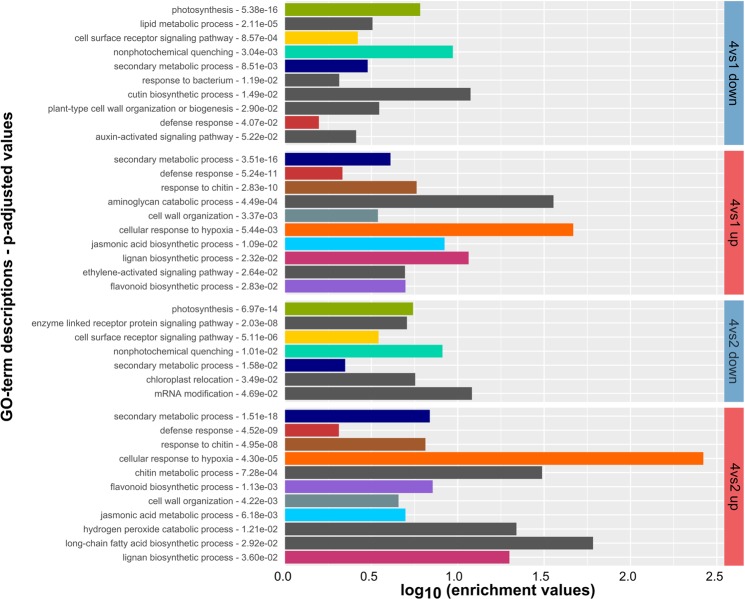
GO term enrichment of lettuce DEGs from interaction zone 1 and 2. Enrichment is calculated from the DESeq output between zone 1 and 2 *vs*. zone 4 (control) with GOrilla and selected terms are depicted with their corresponding false discovery corrected p-values. GO terms that were enriched in two or more groups are indicated by a unique colour, umique terms are presented by grey bars. The full list of enriched GO terms can be found in Supplementary Table S4.

**Figure 4:**
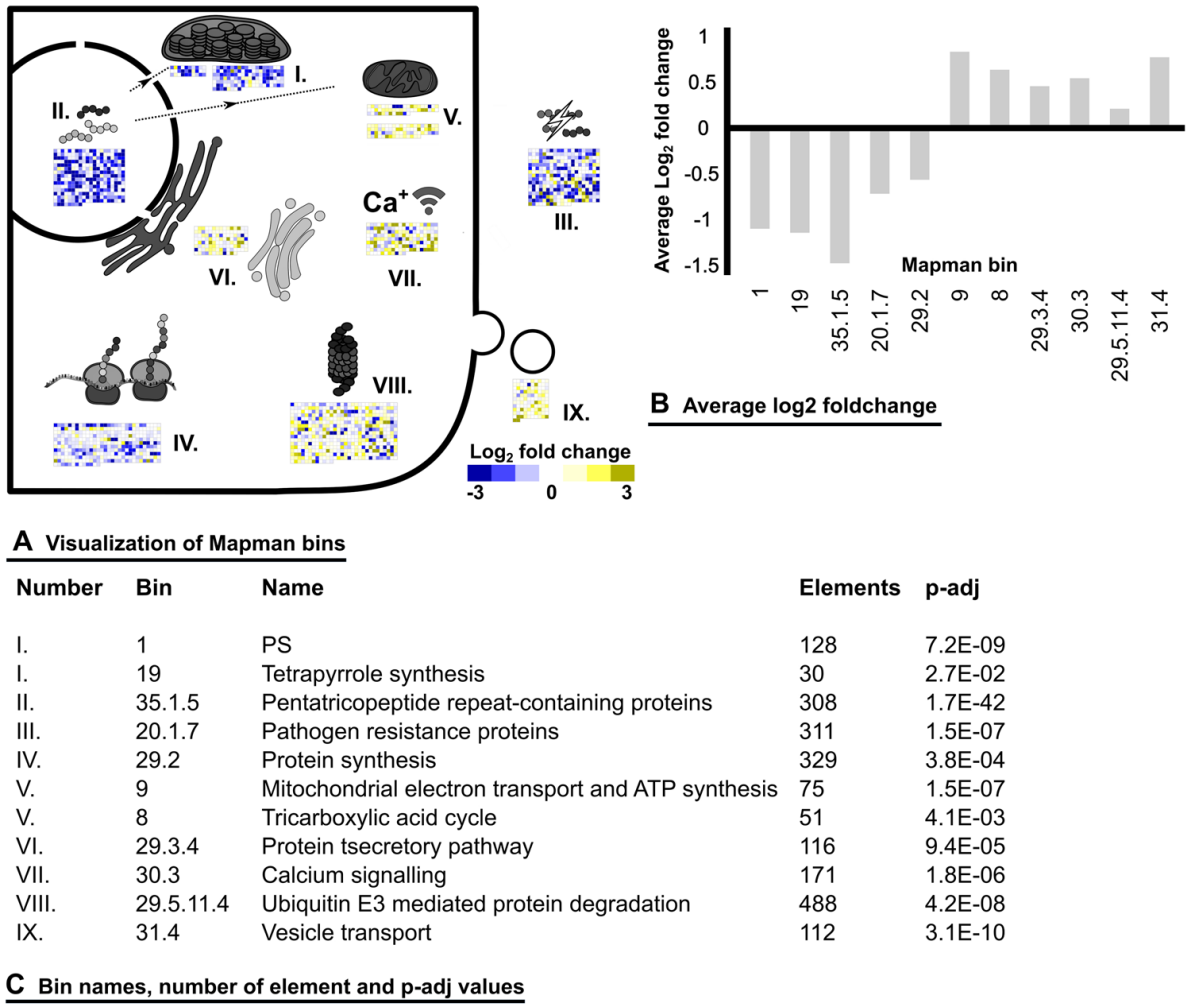
Mapman analysis between the symptomless interaction zone 1 and zone 2 with light discoloration. Fold-changes calculated by DESeq were depicted with Mapman. For fold-changes, a clipping was set at an absolute change of Log_2_ 3 and p-adj values are based on FDR corrected Wilcoxon ranked sum test. (**A**) Visual representation of selected Mapman bins. (**B**) Calculated average Log_2_ fold-changes of Mapman bins. (**C**) Names of depicted Mapman bins, number of elements and p-adj values.

To investigate the functional context of the DEGs between the interaction zones, functional enrichment analyses based on GO terms and Mapman assignments were used (Figs 3 and 4).

Initially, global GO term enrichments in interaction zones 1 and 2 were determined with the software packages GOrilla and ReviGO (Fig. 3). Noteworthy are the enrichments of the terms for defence response, incompatible interaction and the response to chitin, among the up-regulated transcripts. The down-regulated transcripts are enriched in GO terms mostly related to photosynthesis and energy metabolism. Differences between zone 1 and zone 2 are not very prominent, based on this analysis. Nevertheless, some enriched GO term functions are present in zone 1 that are not within zone 2 and *vice versa*. For example, water transport, cell wall modification and response to stimuli are present in zone 1 and long-chain fatty acid biosynthetic process, hydrogen peroxide metabolic process and response to oxygen levels are present in zone 2. A few GO terms are found within both, the up- as well as the down-regulated transcripts.

In addition, enrichment of GO terms was calculated for the Upset groups described above (Fig. 2). This analysis was intended to investigate the functional relationships between the interaction zones. The terms in common between all three interaction zones (group 3 and 16 in Fig. 2) include heme binding, chitinase activity, shikimate metabolic process and chorismate biosynthetic process among the up-regulated transcripts. Chorismate is an important intermediate for plant metabolites like aromatic amino acids and salicylic acid. Several response and synthesis pathways for plant hormones like jasmonate, auxin and cytokinin are down-regulated. This group however is dominated by terms related to photosynthesis. Among the DEGs specific for the symptomless zone 1 (Group 4 and 17 in Fig. 2), the terms for defence response by callose deposition, response to jasmonic acid and ethylene are enriched among the up-regulated DEGs. Down-regulated terms are related to induced systemic resistance (ISR), wax biosynthesis, cell cycle and auxin signalling. Unique to zone 2 (Group 1 and 18 in Fig. 2) are, GO terms that are related to the proteasome and to ATP synthase among the up-regulated DEGs. Down-regulated terms included negative gravitropism and ribosomal biogenesis. Response to chitin and negative regulation of programmed cell death are shared between zone 1 and 2 (Group 2 and 15 in Fig. 2) considering up-regulated genes. Down-regulated terms include many GOs related to the chloroplast and ribosome. A complete list of GO-terms enriched within the Upset groups is included in the Supplementary Table S5.

Subsequently, the transcriptional differences between interaction zone 1 and 2 were investigated and visualized with Mapman based on Mercator annotation of the transcriptome (Fig. 4). This approach revealed increase of transcript abundances in the functional bins related to vesicle transport, protein degradation, mitochondrial energy metabolism and calcium signalling within interaction zone 2. Bins with reduced abundances included protein synthesis, photosynthesis, pathogenesis related-proteins (PR-proteins) and pentatricopeptide (PPR) repeat-containing proteins. PPR containing proteins are suggested to interact with mitochondria and chloroplasts and emulate the function of promotors. These results are also supported by KEGG mapping of DEGs (Supplementary Fig. S3).

### Differential expression of PRG candidates and WRKY transcription factors

**Figure 5:**
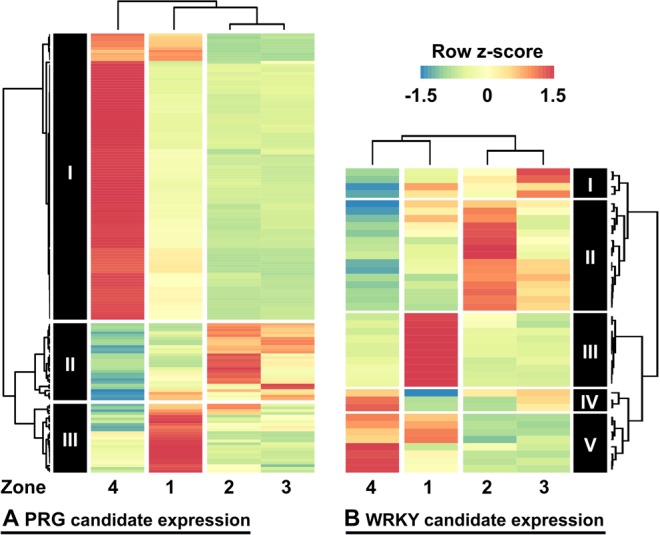
(**A**) Differential expression of pathogen receptor gene (PRG) candidates. (**B**) Differential expression of WRKY transcription factor candidates. Only significantly differentially expressed transcripts are depicted, as identified with DESeq. The heat map colouring depicts row z-score values calculated from RPKM values.

In a previous publication reporting on the lettuce cv. Tizian genome, a subset of 291 pathogen receptor gene candidates (PRG) associated with various pathogens was found within the genome^17^. Of these, 157 PRG candidates were significantly differentially expressed in response to interaction with *R. solani*. The expression patterns within the four sample zones revealed three defined clusters (Fig. 5A). Putative annotations were deduced by a comparison of the protein sequences with the NCBI database nr and BLAST^44^. Cluster I is dominated by transcripts with high abundances in zone 4 and cluster III is dominated by transcripts that are overrepresented in zone 1. In contrast, cluster II comprises transcripts that are abundant in both zone 2 and 3. Zone 2 and 3 are most similar to each other and zone 4 and 1 are more dissimilar, indicating the importance of the R-gene expression pattern shift between zone 1 and 2.

Likewise, the expression patterns of putative WRKY transcription factors, important regulators of plant defences^45^, were analysed by clustering (Fig. 5B). Here the strongest cluster (cluster III) is dedicated to interaction zone 1. This observation indicates an important role for WRKY transcription factors in regulation of the local defence response. The full list of PRG candidates and candidate WRKY transcription factor expression levels is documented in Supplementary Table S6 and 7.

### The lettuce/*R. solani* AG1-IB interaction zones show significant shifts in metabolite compositions

**Figure 6:**
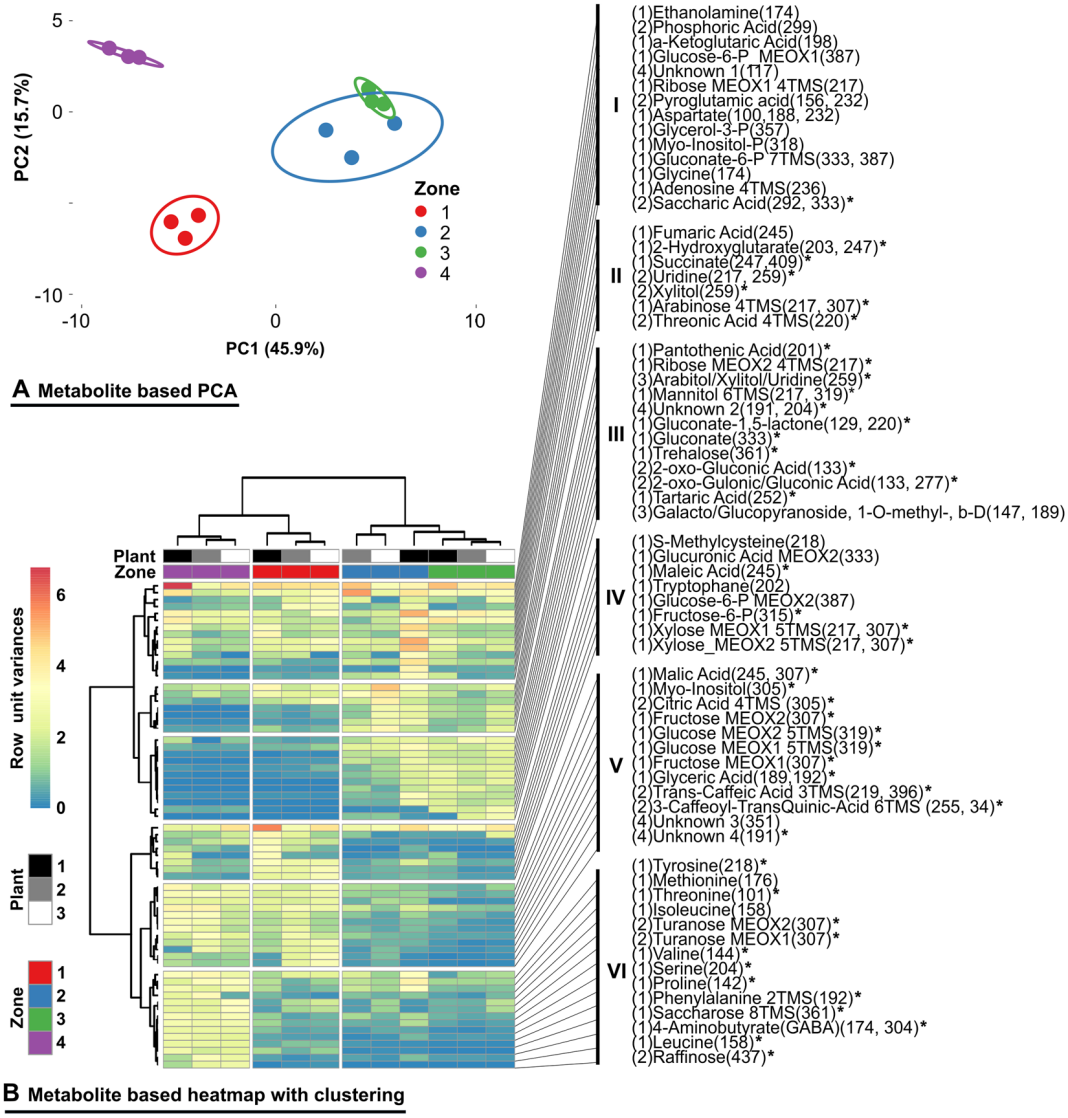
Abundances of selected metabolites within the interaction zones. (**A**) PCA plot based on metabolite abundances. (**B**) Metabolite based heat map; unite variance scaling was applied on rows and Ward clustering was used for rows and columns^31^. The designations of single metabolites include the indicator of the level of identification within the first brackets, according to the reporting standards as proposed by the Metabolomics Standards Initiative^43^ ranging from (1): identified by the measurement of the chemical reference standard, (2): putatively identified by significant database hit, (3): putatively characterized by database hit as compound of a certain chemical class, (4): unknown compound. The chemical name of the compound is accompanied in relevant cases by the information concerning the derivatization status (MEOX, TMS). The last brackets indicate the m/z values, which have been used for the identification and the integration of the signals corresponding to the given metabolite. The asterisks indicate significance as tested with ANOVA. The complete dataset can be found in Supplementary Table S8 as well as an enlarged depiction of heatmap B in Supplementary Fig. 2.

To add a second level of biologically relevant information, the effect of *R. solani* infection upon the metabolite compositions within the analysed interaction zones has been measured by means of GC-MS (Fig. 6). PCA and heat map cluster analysis revealed, as already shown by the transcriptome analysis, that interaction zones 2 and 3 are more similar, whereas the zones 1 and 4 form individual clusters (Fig. 6A). Cluster I mostly comprises metabolites that do not significantly change in the course of the experiment. Cluster II is dominated by metabolites that can be assigned to components of the cell wall or the central metabolism that have an increased abundance within interaction zone 2. Cluster III metabolites are most abundant in the advanced phase of interaction represented by zone 3. Tartaric acid, accumulating in zone 3, is a decomposition product of ascorbate (vitamin C) catabolism with documented fungicidal properties^46^. The metabolites included in cluster IV increase in abundance in interaction zone 1 and decrease again in zones 2 and 3. Glucuronic acid and xylose are both part of hemicellulose and S-methylcystein which play a role as precursors for alexine synthesis. The clusters V and VI comprise metabolites with reduced abundances in zone 2 and 3. Metabolites from cluster VI have lower abundances in all of the interaction zones compared to zone 4. This cluster consists of amino acids and carbohydrates including the non-metabolizable sucrose-analog turanose.

## Discussion

The experimental approach described in this study provides a novel perspective on the lettuce/*Rhizoctonia* pathosystem. Through sampling of lettuce leaf tissue at different distances from the *R. solani* AG1-IB inoculation site as the defining variable, we were able to distinguish three phases defined by their transcriptional patterns as response to interacting *R. solani* hyphae. Hereby, the natural situation involving neighbouring tissues at different levels of interaction at the same time-point were analyzed at the transcriptional level. Regarding the systemic response, we assumed that the different interaction zones cannot be differentiated, whereas intensity of the local interaction increases by decreasing proximity to the inoculation site. The large numbers of detected DEGs as well as the observed metabolome composition shifts verified the relevance of the selected zones since they represent distinguishable interaction phases. The magnitude of both, the transcriptomic as well as the metabolomic changes is another statement to the complexity of plant-host/pathogen interactions. Furthermore, our analysis revealed that in the transition from zone 2 to 3 only relatively few transcriptional changes were observed. This correlates with previous findings regarding the *R. solani* transcriptional response in the same experimental setting^12^. There, it was concluded that most if not all lettuce tissue is diseased in zone 3^12^. Retrospectively, more than 500 DEGs were found to be up-regulated in *R. solani* between zone 2 and 3. Concerning the lettuce transcriptional response, we mainly focused on the interaction zones 1 and 2.

### Reduced transcription of pentatricopeptide genes in zone 1 and 2

Among the identified DEGs, pentatricopeptide (PPR) genes are generally down-regulated in interaction zones 1 and 2, but most prominently in zone 2. PPRs are editors and possible regulators of organelle RNA transcription. Several studies have reported on the negative effects of downregulation of PPRs on defence in plants^3,47–49^. For example, Laluk *et al*.^49^ observed an increased susceptibility of *Arabidopsis* to *B. cinerea* in a knockout mutant of PNG, a PPR protein that is localized in the mitochondrion. Interestingly, these authors also described an increase in abscisic acid (ABA) sensitivity of the mutant; ABA signalling was among the enriched GO terms in our analysis as well.

### Reduction of transcripts related to the chloroplast in zone 1 and 2

In our analysis, we found a gradual down-regulation of many DEGs related to the chloroplast between zones 1 and 2. The chloroplast is crucial in the signalling cascade for PAMP perception and synthesis of the phytohormones SA, JA and ABA as well as production of ROS^50–53^. Previous studies have also reported on down-regulation of photosynthesis genes after perception of a PAMP signal^54^. With this, the host probably aims for limiting the availability of carbohydrates to the pathogen. This strategy probably is effective in the defence reaction against biotrophs. However, our metabolic analysis revealed that glucose and fructose among other sugars are still readily available in zone 1 of the lettuce/*R. solani* interaction. A second possibility is that the host dedicates its resources completely to the synthesis of defence products, up to an extent that it is no longer feasible to maintain the process of photosynthesis. Finally, downregulation of chloroplast related genes and especially those related to non-photochemical quenching promote the production of ROS. The GO term for non-photochemical quenching was also enriched among the downregulated DEGs in this experiment. Specifically, a candidate gene for Photosystem II 22 kDA protein (PsbS, TCONS_00021596_g.24734) was downregulated in the *R. solani* three interaction zones.

### Increased transcription of calcium signalling related genes potentially involved in ROS synthesis

Calcium signalling is induced in interaction zone 1 and even more within zone 2. Within plants, calcium signalling plays a role in various biological processes including defence reactions and the hypersensitive response. Zhang *et al*. also reported on up-regulation of calcium signalling related transcripts in rice 24 hours after inoculation with *R. solani* AG1-IA^55^. Among the induced genes in lettuce, there are various candidates encoding calcium dependent protein kinases (CDPKs). These can have various functions such as hormonal signalling, stomatal closure, oxidative burst and induction of cell death^56^. In our data, we observed pathogen induced up-regulation of candidate genes for CDPK1, CDPK4, CDPK7 and CPDK26 like proteins. In *Arabidopsis*, AtCPK1 is involved in camalexin accumulation, SA signalling and in SA-independent ROS activation together with CDPK4 in *Arabidopsis* and *Solanum tuberosum*^57^. The latter is achieved through Respiratory Burst Oxidase Homologs (RBOHs). Two candidate genes, namely RBOHE and RBOHA, were progressively induced from zone 1 to 2 (TCONS_00026252_g.30371, TCONS_00009774_g.11091) and might be involved in SA independent activation of ROS-induced PCD^58^. Several more indicators of ROS-induced programmed cell death (PCD) involvement within the lettuce/*R. solani* pathosystem were found, like the up-regulation of a metacaspase-1-like encoding transcript (TCONS_00035876_g.40713) and general transcripts related to cell vesicle transport and the proteasome. Contradictory to these findings, the GO term for negative regulation of PCD (GO:0043069) was upregulated within interaction zones 1 and 2. Considering the experimental setup and the contradictory result regarding PCD, we hypothesize that upon infection by *R. solani*, negative regulation of PCD is systemically induced in viable tissue (zone 1 and 2). In contrast, induced PCD and hypersensitive response (HR) occurs as a localized event, limited to single cells directly interacting with fungal hyphae.

### Co-transcription of PRG candidates between the interaction zones

In a previous study, we described a sub-set of putative lettuce cv. Tizian PRG candidates associated with various pathogens^17^. Transcriptome data of these type of genes is challenging to interpret in a non-model species like lettuce due to interference and crosstalk between various defence pathways. In this study, a large cluster of co-transcribed genes, specific for zone 4, was found to contain candidates encoding well documented R-genes like Xa21 and the TMV-resistance protein gene^59,60^. As expected, these are down-regulated within the interaction zones since they are specific for bacterial and viral pathogens. Less expected was the down-regulation of genes whose gene products may assist in the defence of necrotrophic pathogens like those encoding LRR receptor-like serine/threonine-protein kinase ERECTA (TCONS_00025278_g.29229 and TCONS_00036128_g.41007) proteins, who are involved in regulation of immune responses^61^. Furthermore, putative Wall Associated Kinase 1 (WAK1) candidate transcripts are more abundant in zone 1, whereas WAK2 related transcripts were more abundant in zones 2 and 3. A similar distribution was found for several receptor-like protein CLAVATA1-like and CLAVATA2-like encoding transcripts (TCONS_00008091_g.9105, TCONS_00002540_g.2858 and TCONS_00029043_g.33326). In *Arabidopsis*, WAK1 is a receptor for oligo-galacturonide DAMPs that can be induced by either JA or SA and can directly induce ROS defence reactions^62^. Underlining the importance of receptors and specifically MAPs during the interaction, we found two candidate genes for leucine-rich repeat (LRR) receptor-like serine/threonine-protein kinase (EFR) guard proteins that were upregulated in all three interaction zones^63^.

### Comparison of GO term enrichment between lettuce responses to the pathogens *R. solani* and *Botrytis cinerea*

De Cremer *et al*.^22^ previously reported on the response of lettuce to interaction with *Botrytis cinerea*. Like *R. solani*, *B. cinerea* is considered to represent a pathogen featuring a general necrotrophic lifestyle. Hence, we expected that these two pathosystems probably share a high degree of similarity regarding progression of pathogenic interaction. However, comparison of the corresponding global GO term analyses revealed that the lettuce response to *B. cinerea* at 48 h as reported by De Cremer *et al*. has only 14% of the up-regulated and 38% of the down-regulated enriched GO terms in common with the *R. solani* pathosystem interaction zone 2. This is most likely a result of the specific responses of lettuce towards the two pathogens and methodical differences between both interaction experiments. The shared down-regulated GO terms are almost exclusively dedicated to the photosystem machinery. An exception is the transmembrane receptor protein tyrosine kinase signalling pathway. The enrichment of this group appears to be caused by the large cluster of down-regulated R-gene candidates featuring receptor related functions, that were differentially expressed in our experiment. Among the shared up-regulated GO terms, we found GOs related to phenylpropanoid, chitin, and lignin responses to fungi and systemic acquired resistance (SAR). Additionally, terms related to the plant hormones JA, ET, ABA and AUX are enriched in both pathosystems. Although it may be suggested that these GOs are part of a universal response towards fungal pathogens. It should be kept in mind that both pathosystems involve compatible interactions that do not result in successful defence against their respective pathogens.

### Transcription of SA and JA signalling genes

Recently Kouzai *et al*.^64^ reported on the discovery of Salicylic acid (SA) dependent resistance of *Oryza sativa* (rice) and *Brachypodium distachyon* towards *R. solani*, suggesting the existence of a pseudo biotrophic phase during the interaction with these two host species. The importance of SA mediated defences plant defences against *R. solani* was further underlined by Genzel *et al*.^65^. Interestingly, no enriched GO terms related to SA signalling were found to be enriched in our dataset. On the contrary, we observed induction of genes that might trigger fine-tuning of the hormonal balance between SA and JA. For example, a putative transcript for Jasmonate Resistant 1 (JAR1) was continuously lower abundant during the interaction (TCONS_00020356_g.23215). JAR1 belongs to the group of auxin responsive genes and synthesizes jasmonate-isoleucine, the most active form of JA. JAR1 and Coronatine-insensitive protein 1 (COI1, TCONS_00010993_g.12484) are necessary for *Arabidopsis* resistance against *B. cinerea* and induce sensitivity towards *F. graminearum* by attenuation of SA signalling^66^. Presence of Non-expressor of PR gene 1 (NPR1), a regulator of SA signalling and SAR, masks the latter phenotype. The TCONS_00016219_g.18411 NPR1 candidate gene is continuously expressed throughout the experiment. In addition, a putative CYP94B3 encoding transcript (TCONS_00024974_g.28887) is up-regulated in zone 1 and 2. CYP94B3 catabolizes and inactivates jasmonate-isoleucine. Additionally, a set of 45 transcripts related to auxin-activated signalling (GO:0009734) is down-regulated.

### WRKY transcription factors

Further regulatory elements with highly specific induction between the interaction zones are the WRKY transcription factor family members. With fold-changes of up to 60, it can be assumed that these are of great importance during the lettuce/*R. solani* interaction. WRKYs play essential roles in plants including biotic and abiotic defences, development and senescence^45^. Although WRKYs have been studied for many plant species, only little information is available for lettuce WRKYs. Apart from activation of plant defences, *Hordeum vulgare* WRKY1 and WRKY2 homologs for example are involved in regulation of PTI and ETI responses^67^, whereas two close homologs of rice WRKY45 either regulate SA or JA levels^68^. Future research on lettuce WRKYs should elucidate their function in pathogen interactions and hormone homeostasis.

### Metabolite profiles are distinctive for each interaction zone

Regarding the metabolite analysis, we should mention that in contrast to the previously discussed transcriptome analysis, most metabolites cannot be attributed to one of the two interaction partners definitively. Combining the knowledge gained from the transcriptomes and comparison with the blank and in relation to the level of interaction, these profiles can be interpreted.

One of the most interesting compounds identified in the metabolite analysis is the non-metabolizable sucrose-analog turanose. It is involved in signal transduction and priming of defence in *Lycopersicon peruvianum* (tomato), *Zea mays* (maize) and *Oryza sativa* (rice)^69,70^. Several studies report on a reduction of the turanose content after plant infection with *R*. *solani*, most likely because fungi unlike plants are capable to metabolize turanose^70,71^. In contrast, co-inoculation with *Bacillus amyloliquefaciens* resulted in a turanose concentration comparable to the control samples and it was suggested that this adjustment is mechanistic in plant priming. We propose a slightly modified hypothesis where not the absolute concentration but the change in concentration of turanose is able to prime plant defence. Further compounds with reduced concentrations during interaction comprised the amino acids tyrosine, methionine, threonine, isoleucine, valine, serine, proline, phenylalanine, 4-aminobutyrat and leucine. The decrease of these compounds indicates their importance during the defence. KEGG pathway mapping of the transcriptome data indicated clear up-regulation within the KEGG pathways for phenylalanine, tyrosine, tryptophan, valine, leucine and isoleucine synthesis. In addition, the degradation of cysteine, methionine, valine, leucine, isoleucine and sphingolipid and the synthesis of phenylpropanoid also appeared to be up-regulated which may result in a decrease of the aforementioned compounds despite the up-regulation of the corresponding synthesis pathways. Subsequently, several intermediates of the phenylpropanoid pathway decreased in concentration in interaction zone 2. Integrating this information and the transcriptome mapping onto the phenylpropanoid pathway suggests a flux directed towards the production of synapaldehyde and synapoyl-CoA representing precursors of lignin. Malate, citric acid, glucose and fructose fuel the glycolysis and oxidative phosphorylation pathways and satisfy the increased energy demand. In addition, glucose probably is utilized for cellulose synthesis. Glucuronic acid and xylose show a small peak in concentration within interaction zone 1. These are both possible components of hemicellulose and S-methylcysteine; the latter is a possible precursor for alexine synthesis. Both compounds are increased in concentration in zone 2, Arabinose and xylitol are most likely degradation products of *R. solani* induced cell wall lysis. Alternatively, it is possible that downstream synthesis of hemi-cellulose is restrained, causing the accumulation of precursors components. Threonic acid and tartaric acid, decomposition products of ascorbate, increase in concentrations within zone 2. Although ascorbate concentrations were not directly measured, it can be assumed, based on transcriptome data and the above mentioned results, that the ascorbate concentration is reduced, a status that has been linked to induction of senescence^72^. Furthermore, vitamin C is an important anti-oxidative protecting agent against ROS and tartaric acid cannot be metabolised by fungi.

### Concluding remarks

In this study, we have demonstrated that inoculation of lettuce cv. Tizian with *R. solani* AG1-IB causes large-scale reprogramming of the transcriptome and metabolome in lettuce leaf tissue. Our data suggest that the role of lettuce in the interaction is not as passive as was previously assumed. Analysis of the metabolite profiles and the differentiated patterns of PRG transcript levels between the three interaction zones very much corroborates active defence efforts of lettuce. The defence reaction of lettuce has more in common with a guarded retreat, where spread of the fungus is minimized or retarded, instead of an offensive defence approach. In the introduction, we postulated that an SA mediated early response can be beneficial during the initial phase of interaction. Eventually, though, during the infective phase of interaction *R. solani* is a necrotrophic pathogen and JA mediated defences should be dominant. Therefore, the consistent down-regulation of JAR1 and several auxin responsive genes was very surprising, as was the lack of enrichment for SA responsive genes. The apparent improper regulation of JA and SA signalling, adapted to the specific interaction zones, appears to be a clear disadvantage and might weaken the effectiveness of the lettuce local defence response. Alternatively, *R. solani* may actively interfere with host hormonal signalling, deregulating the defence response. In the future, more research is needed to confirm these findings, including experimental work with whole plant interaction models. As such, future work should address identification of elicitors responsible for inducing lettuce PCD/senescence within this pathosystem. Furthermore, hormone levels should be measured directly to analyse their influence and correlation with the transcription profiles that were described here and lettuce PRG and WRKY candidate gene functions have to be elucidated.

## Supplementary information

Lettuce and R. solani leaf interaction model

Supplementary figures

Supplementary Dataset 3

## Data Availability

The sequencing raw data for all libraries is available from EBI ArrayExpress, accession E-MTAB-4762.

## References

[1] Grosch R, Schneider, Kofoet A, Feller C (2011). Impact of continuous cropping of lettuce on the disease dynamics of bottom rot and genotypic diversity of *rhizoctonia solani* AG 1-IB. J. Phytopathol..

[2] Glazebrook J (2005). Contrasting mechanisms of defense against biotrophic and necrotrophic pathogens. Annu. Rev. Phytopathol..

[3] Laluk K, Mengiste T (2010). Necrotroph attacks on plants: wanton destruction or covert extortion?. Arabidopsis Book.

[4] Horbach R, Navarro-Quesada AR, Knogge W, Deising HB (2011). When and how to kill a plant cell: Infection strategies of plant pathogenic fungi. J. Plant Physiol..

[5] Mengiste T (2012). Plant Immunity to Necrotrophs. Annu. Rev. Phytopathol..

[6] Ogoshi Akira (1996). Introduction — The Genus Rhizoctonia. Rhizoctonia Species: Taxonomy, Molecular Biology, Ecology, Pathology and Disease Control.

[7] Boller T, Felix G (2009). A Renaissance of Elicitors: Perception of Microbe-Associated Molecular Patterns and Danger Signals by Pattern-Recognition Receptors. Annu. Rev. Plant Biol..

[8] Wang X, Jiang N, Liu J, Liu W, Wang G-L (2014). The role of effectors and host immunity in plant-necrotrophic fungal interactions. Virulence.

[9] Thomma BPHJ, Nürnberger T, Joosten MHAJ (2011). Of PAMPs and Effectors: The Blurred PTI-ETI Dichotomy. Plant Cell.

[10] Thatcher LF, Manners JM, Kazan K (2009). *Fusarium oxysporum* hijacks COI1-mediated jasmonate signaling to promote disease development in Arabidopsis. Plant J..

[11] Godoy-Lutz G, Steadman JR, Higgins B, Powers K (2003). Genetic Variation Among Isolates of the Web Blight Pathogen of Common Bean Based on PCR-RFLP of the ITS-rDNA Region. Plant Dis..

[12] Verwaaijen, B. *et al*. The *Rhizoctonia solani* AG1-IB (isolate 7/3/14) transcriptome during interaction with the host plant lettuce (*Lactuca sativa* L.). *PLoS One* **12** (2017). 10.1371/journal.pone.0177278 PMC5423683 28486484

[13] Wibberg D (2014). Transcriptome analysis of the phytopathogenic fungus *Rhizoctonia solani* AG1-IB 7/3/14 applying high-throughput sequencing of expressed sequence tags (ESTs). Fungal Biol..

[14] Wibberg D (2015). Improved genome sequence of the phytopathogenic fungus *Rhizoctonia solani* AG1-IB 7/3/14 as established by deep mate-pair sequencing on the MiSeq (Illumina) system. J. Biotechnol..

[15] Wibberg D (2013). Establishment and interpretation of the genome sequence of the phytopathogenic fungus *Rhizoctonia solani* AG1-IB isolate 7/3/14. J. Biotechnol..

[16] Wibberg Daniel, Rupp Oliver, Blom Jochen, Jelonek Lukas, Kröber Magdalena, Verwaaijen Bart, Goesmann Alexander, Albaum Stefan, Grosch Rita, Pühler Alfred, Schlüter Andreas (2015). Development of a Rhizoctonia solani AG1-IB Specific Gene Model Enables Comparative Genome Analyses between Phytopathogenic R. solani AG1-IA, AG1-IB, AG3 and AG8 Isolates. PLOS ONE.

[17] Verwaaijen B (2018). Assembly of the *Lactuca sativa*, L. cv. Tizian draft genome sequence reveals differences within major resistance complex 1 as compared to the cv. Salinas reference genome. J. Biotechnol..

[18] Christopoulou M (2015). Genome-Wide Architecture of Disease Resistance Genes in Lettuce. G3 (Bethesda)..

[19] McHale LK (2009). The genomic architecture of disease resistance in lettuce. Theor. Appl. Genet..

[20] Truco MJ (2013). An Ultra High-Density, Transcript-Based, Genetic Map of Lettuce. G3 (Bethesda)..

[21] Simko Ivan, Ochoa Oswaldo E., Pel Mathieu A., Tsuchida Cayla, Font i Forcada Carolina, Hayes Ryan J., Truco Maria-Jose, Antonise Rudie, Galeano Carlos H., Michelmore Richard W. (2015). Resistance to Downy Mildew in Lettuce ‘La Brillante’ is Conferred byDm50Gene and Multiple QTL. Phytopathology.

[22] De Cremer, K. *et al*. RNAseq-based transcriptome analysis of *Lactuca sativa* infected by the fungal necrotroph Botrytis cinerea. *Plant*. *Cell Environ*., 10.1111/pce.12106 (2013). 10.1111/pce.12106 23534608

[23] Grube RC (2003). Characterization and genetic analysis of a lettuce (*Lactuca sativa* L.) mutant, weary, that exhibits reduced gravitropic response in hypocotyls and inflorescence stems. J. Exp. Bot..

[24] Meyers BC (1998). The major resistance gene cluster in lettuce is highly duplicated and spans several megabases. Plant Cell.

[25] Rezzonico, F., Rupp, O. & Fahrentrapp, J. Pathogen recognition in compatible plant-microbe interactions. *Sci*. *Rep*. 1–12, 10.1038/s41598-017-04792-5 (2017). 10.1038/s41598-017-04792-5 PMC5526865 28743967

[26] Copley, T. R., Aliferis, K. A., Kliebenstein, D. J. & Jabaji, S. H. An integrated RNAseq- 1 H NMR metabolomics approach to understand soybean primary metabolism regulation in response to Rhizoctonia foliar blight disease. 1–18, 10.1186/s12870-017-1020-8 (2017). 10.1186/s12870-017-1020-8 PMC5408482 28449662

[27] Stefańczyk, E., Sobkowiak, S., Brylińska, M. & Śliwka, J. Expression of the Potato Late Blight Resistance Gene Rpi-phu1 and *Phytophthora infestans* Effectors in the Compatible and Incompatible Interactions in Potato. *Phytopathology* 740–748 (2017). 10.1094/PHYTO-09-16-0328-R 28134594

[28] Kim D (2013). TopHat2: accurate alignment of transcriptomes in the presence of insertions, deletions and gene fusions. Genome Biol..

[29] Hilker R (2014). ReadXplorer–visualization and analysis of mapped sequences. Bioinformatics.

[30] Hilker Rolf, Stadermann Kai Bernd, Schwengers Oliver, Anisiforov Evgeny, Jaenicke Sebastian, Weisshaar Bernd, Zimmermann Tobias, Goesmann Alexander (2016). ReadXplorer 2—detailed read mapping analysis and visualization from one single source. Bioinformatics.

[31] Metsalu T, Vilo J (2015). ClustVis: A web tool for visualizing clustering of multivariate data using Principal Component Analysis and heatmap. Nucleic Acids Res..

[32] Boettiger C (2015). An introduction to Docker for reproducible research. ACM SIGOPS Oper. Syst. Rev..

[33] Love, M., Anders, S. & Huber, W. *Differential analysis of RNA-Seq data at the gene level using the DESeq2 package* (2013).

[34] Benjamini Y, Hochberg Y (2016). Controlling the False Discovery Rate: A Practical and Powerful Approach to Multiple Testing Author (s): Yoav Benjamini and Yosef Hochberg Source: Journal of the Royal Statistical Society. Series B (Methodological), Vol. 57, No. 1 (1995). Publi. J. R. Stat. Soc..

[35] Lex A, Gehlenborg N, Strobelt H, Vuillemot R, Pfister H (2014). UpSet: Visualization of intersecting sets. IEEE Trans. Vis. Comput. Graph..

[36] Eden E, Navon R, Steinfeld I, Lipson D, Yakhini Z (2009). GOrilla: a tool for discovery and visualization of enriched GO terms in ranked gene lists. BMC Bioinformatics.

[37] Lohse M (2014). Mercator: A fast and simple web server for genome scale functional annotation of plant sequence data. Plant, Cell Environ..

[38] Usadel B (2009). A guide to using MapMan to visualize and compare Omics data in plants: A case study in the crop species, Maize. Plant, Cell Environ..

[39] Kanehisa M, Goto S (2000). KEGG: Kyoto Encyclopedia of Genes and Genomes. Nucleic Acids Res..

[40] Tanabe M, Sato Y, Morishima K, Furumichi M, Kanehisa M (2016). KEGG: new perspectives on genomes, pathways, diseases and drugs. Nucleic Acids Res..

[41] Kanehisa M, Sato Y, Furumichi M, Morishima K, Tanabe M (2019). New approach for understanding genome variations in KEGG. Nucleic Acids Res..

[42] Moriya Y, Itoh M, Okuda S, Yoshizawa AC, Kanehisa M (2007). KAAS: An automatic genome annotation and pathway reconstruction server. Nucleic Acids Res..

[43] Sumner LW (2007). Proposed minimum reporting standards for chemical analysis. Metabolomics.

[44] Altschul SF, Gish W, Miller W, Myers EW, Lipman DJ (1990). Basic local alignment search tool. J. Mol. Biol..

[45] Rushton PJ, Somssich IE, Ringler P, Shen QJ (2010). WRKY transcription factors. Trends Plant Sci..

[46] Shokri H (2011). Evaluation of inhibitory effects of citric and tartaric acids and their combination on the growth of *Trichophyton mentagrophytes*, *Aspergillus fumigatus*, *Candida albicans*, and *Malassezia furfur*. Comp. Clin. Path..

[47] Park YJ (2014). MicroRNA400-guided cleavage of pentatricopeptide repeat protein mRNAs renders *Arabidopsis thaliana* more susceptible to pathogenic bacteria and fungi. Plant Cell Physiol..

[48] Xing H (2018). Genome-wide investigation of pentatricopeptide repeat gene family in poplar and their expression analysis in response to biotic and abiotic stresses. Sci. Rep..

[49] Laluk K, AbuQamar S, Mengiste T (2011). The *Arabidopsis* Mitochondria-Localized Pentatricopeptide Repeat Protein PGN Functions in Defense against Necrotrophic Fungi and Abiotic Stress Tolerance. Plant Physiol..

[50] Wasternack C, Hause B (2013). Jasmonates: Biosynthesis, perception, signal transduction and action in plant stress response, growth and development. An update to the 2007 review in Annals of Botany. Ann. Bot..

[51] Dempsey DA, Vlot AC, Wildermuth MC, Klessig DF (2011). Salicylic Acid Biosynthesis and Metabolism. Arab. B..

[52] Nambara E, Marion-Poll A (2005). Abscisic Acid Biosynthesis and Catabolism. Annu. Rev. Plant Biol..

[53] Serrano I, Audran C, Rivas S (2016). Chloroplasts at work during plant innate immunity. J. Exp. Bot..

[54] Göhre V, Jones AME, Sklenář J, Robatzek S, Weber APM (2012). Molecular Crosstalk Between PAMPTriggered Immunity and Photosynthesis. Mol. Plant-Microbe Interact..

[55] Zhang J (2017). Comparative Transcriptome Analyses of Gene Expression Changes Triggered by *Rhizoctonia solani* AG1 IA Infection in Resistant and Susceptible Rice Varieties. Front. Plant Sci..

[56] Boudsocq M, Sheen J (2013). CDPKs in immune and stress signaling. Trends Plant Sci..

[57] Xing T, Wang XJ, Malik K, Miki BL (2001). Ectopic expression of an *Arabidopsis* calmodulin-like domain protein kinase-enhanced NADPH oxidase activity and oxidative burst in tomato protoplasts. Mol. Plant. Microbe. Interact..

[58] Chen J, Gutjahr C, Bleckmann A, Dresselhaus T (2015). Calcium signaling during reproduction and biotrophic fungal interactions in plants. Mol. Plant.

[59] Song WY (1997). Evolution of the rice Xa21 disease resistance gene family. Plant Cell.

[60] Hehl R (1999). TMV resistance gene N homologues are linked to *Synchytrium endobioticum* resistance in potato. Theor. Appl. Genet..

[61] Jordá, L., Sopeña-torres, S., Escudero, V. & Nuñez-corcuera, B. ERECTA and BAK1 Receptor Like Kinases Interact to Regulate Immune Responses in *Arabidopsis*. **7**, 1–15 (2016). 10.3389/fpls.2016.00897 PMC4923796 27446127

[62] Brutus A, Sicilia F, Macone A, Cervone F, De Lorenzo G (2010). A domain swap approach reveals a role of the plant wall-associated kinase 1 (WAK1) as a receptor of oligogalacturonides. Proc. Natl. Acad. Sci..

[63] Dodds PN, Rathjen JP (2010). Plant immunity: towards an integrated view of plant–pathogen interactions. Nat. Rev. Genet..

[64] Kouzai Y (2018). Salicylic acid-dependent immunity contributes to resistance against *Rhizoctonia solani*, a necrotrophic fungal agent of sheath blight, in rice and *Brachypodium distachyon*. New Phytol..

[65] Genzel F, Franken P, Witzel K, Grosch R (2017). Salicylic acid-related plant defences are systemically induced in potato in response to *Rhizoctonia solani* AG3PT. Plant Pathol..

[66] Makandar R (2010). Involvement of salicylate and jasmonate signaling pathways in Arabidopsis interaction with *Fusarium graminearum*. Mol. Plant. Microbe. Interact..

[67] Shen QH (2007). Nuclear activity of MLA immune receptors links isolate-specific and basal diseaseresistance responses. Science (80)..

[68] Tao Z (2009). A Pair of Allelic WRKY Genes Play Opposite Roles in Rice-Bacteria Interactions. Plant Physiol..

[69] Sinha AK (2002). Metabolizable and non-metabolizable sugars activate different signal transduction pathways in tomato. Plant Physiol..

[70] Ghosh, S., Kanwar, P. & Jha, G. Alterations in rice chloroplast integrity, photosynthesis and metabolome associated with pathogenesis of *Rhizoctonia solani*. *Sci*. *Rep*. **7** (2017). 10.1038/srep41610 PMC5292701 28165003

[71] Srivastava, S., Bist, V., Srivastava, S. & Singh, P. C. Unraveling Aspects of *Bacillus amyloliquefaciens* Mediated Enhanced Production of Rice under Biotic Stress of *Rhizoctonia solani*. **7**, 1–16 (2016). 10.3389/fpls.2016.00587 PMC4858605 27200058

[72] Pavet V (2005). Ascorbic Acid Deficiency Activates Cell Death and Disease Resistance Responses in *Arabidopsis* 1. Society.

