# Supplementary material for: A comprehensive analysis of the Lactuca sativa, L. transcriptome during different stages of the compatible interaction with Rhizoctonia solani
Source: Sci Rep. 2019 May 10;9:7221. doi: 10.1038/s41598-019-43706-5 (PMC6510776; doi:10.1038/s41598-019-43706-5)
Supplement: Supplementary file 2 — Supplementary figures [file 41598_2019_43706_MOESM2_ESM.pdf]

A comprehensive analysis of the *Lactuca sativa*, L. transcriptome during different stages of the compatible interaction with *Rhizoctonia solani*

Bart Verwaaijen[1,2,3], Daniel Wibberg[1], Anika Winkler[1], Rita Zrenner[2], Hanna Bednarz[1], Karsten Niehaus[1], Rita Grosch[2], Alfred Pühler[1], Andreas Schlüter[1]*

[1]Center for Biotechnology, Bielefeld University, Bielefeld, Germany

[2]Leibniz-Institute of Vegetable and Ornamental Crops (IGZ), Großbeeren, Germany

[3]Current address, department of Computational Biology, Bielefeld University, Bielefeld, Germany

Table of contents:

Supplementary Figure S1

Supplementary Figure S2

Supplementary Figure S3

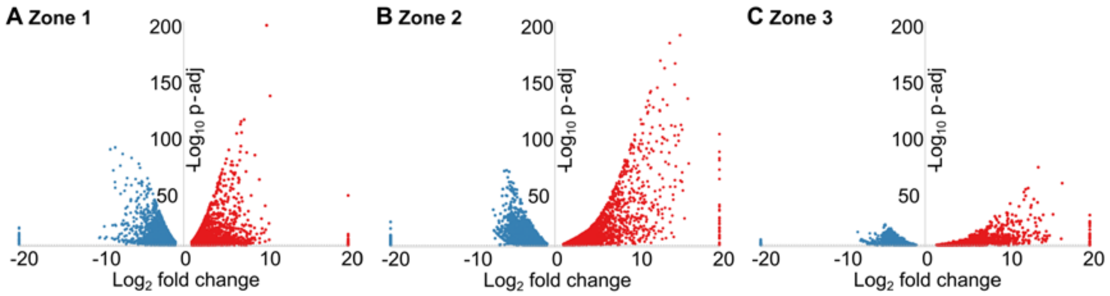

Supplementary Figure S1: DESeq based volcano plots depicting the differentially expressed genes of *L. sativa* between the interaction zones 1, 2 and 3 in comparison to the control (zone 4).

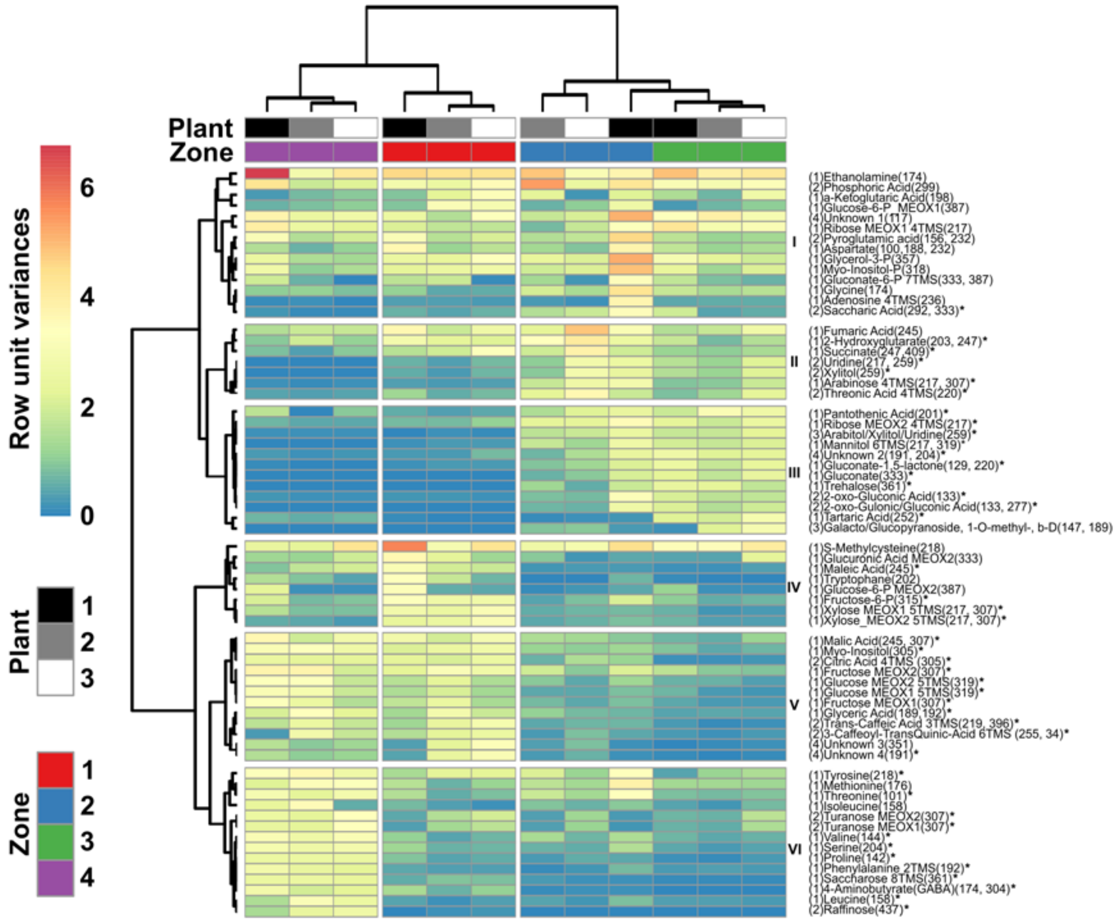

Supplementary Figure S2: Enlarged depiction of Figure 6B. Metabolite based heat map; unite variance scaling was applied on rows and Ward clustering was used for rows and columns[31]. The designations of single metabolites include the indicator of the level of identification within the first brackets, according to the reporting standards as proposed by the Metabolomics Standards Initiative [41] ranging from (1): identified by the measurement of the chemical reference standard, (2): putatively identified by significant database hit, (3) putatively characterized by database hit as compound of a certain chemical class, (4): unknown compound. The chemical name of the compound is accompanied in relevant cases by the information concerning the derivatization status (MEOX,TMS). The last brackets indicate the m/z values, which have been used for the identification and the integration of the signals corresponding to the given metabolite. The asterisks indicate significance as tested with ANOVA.

KAAS Kegg mapping of *L. sativa* cv. Tizian DEG's

Only DEG's with p-adjusted values of 0.05 or smaller and absolute fold changes of 2 or higher are depicted

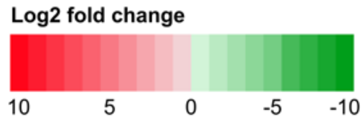

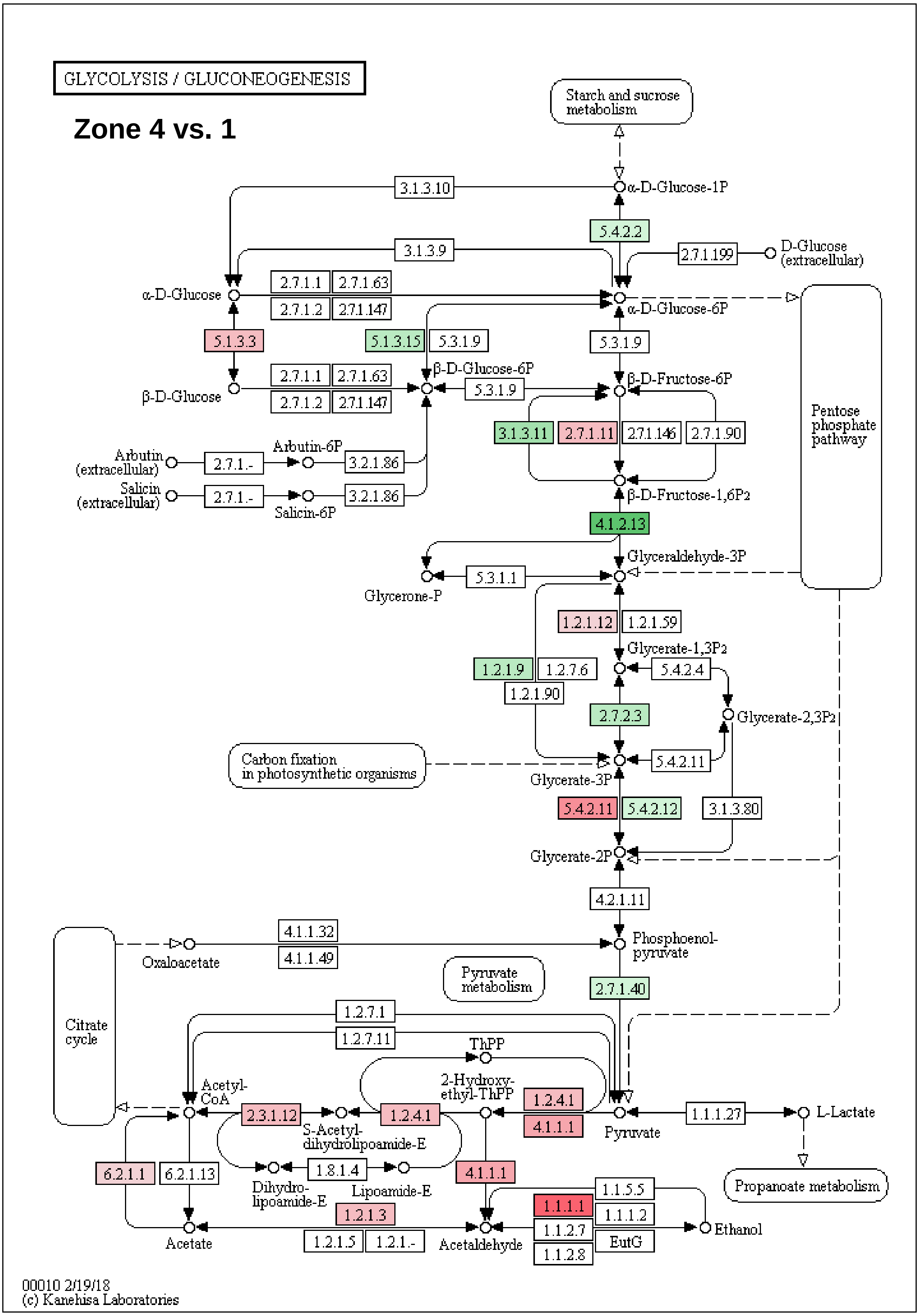

KAAS Kegg mapping of *L. sativa* cv. Tizian DEG's

Only DEG's with p-adjusted values of 0.05 or smaller and absolute fold changes of 2 or higher are depicted

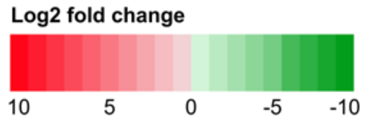

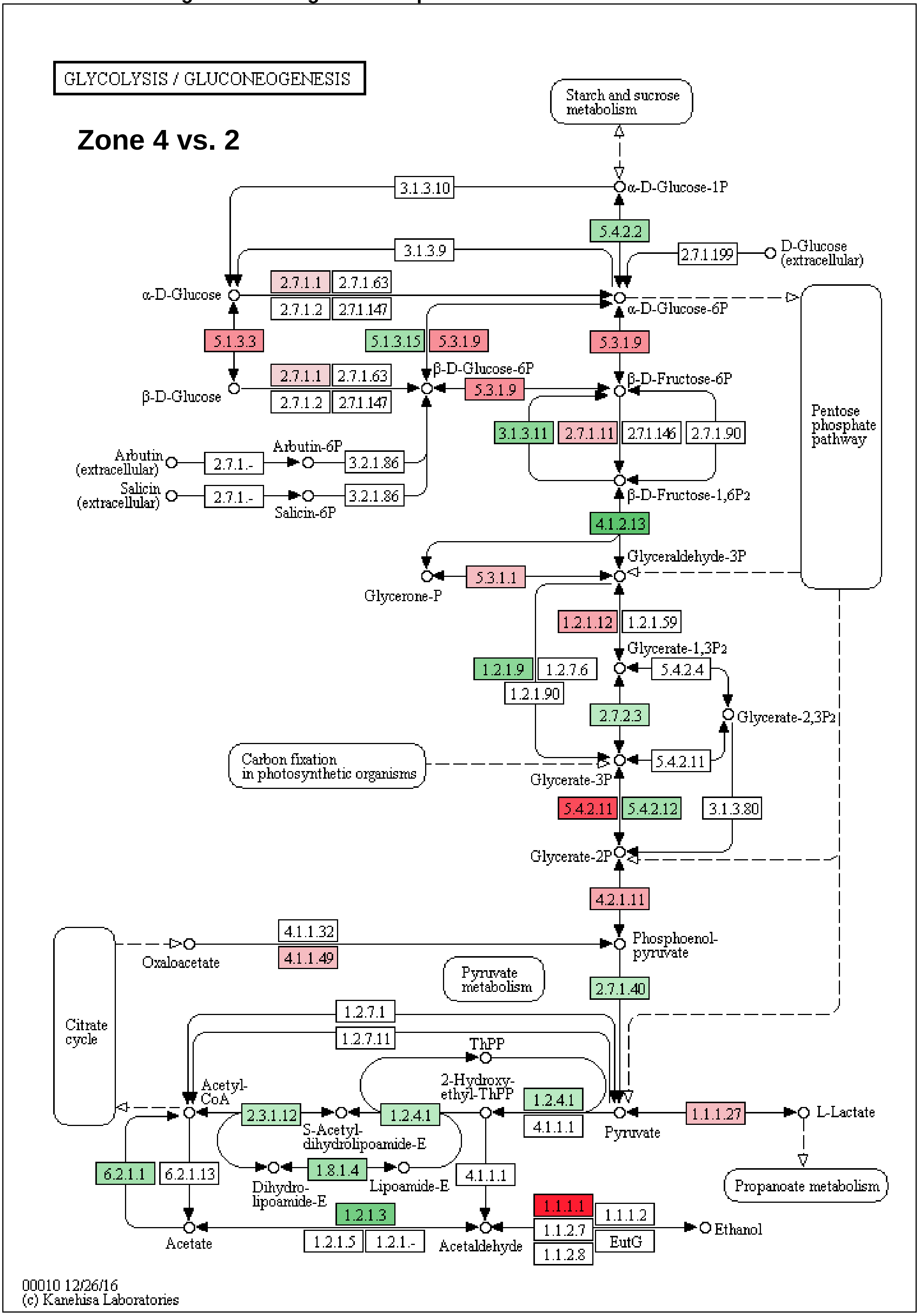

KAAS Kegg mapping of *L. sativa* cv. Tizian DEG's

Only DEG's with p-adjusted values of 0.05 or smaller and absolute fold changes of 2 or higher are depicted

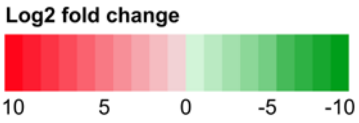

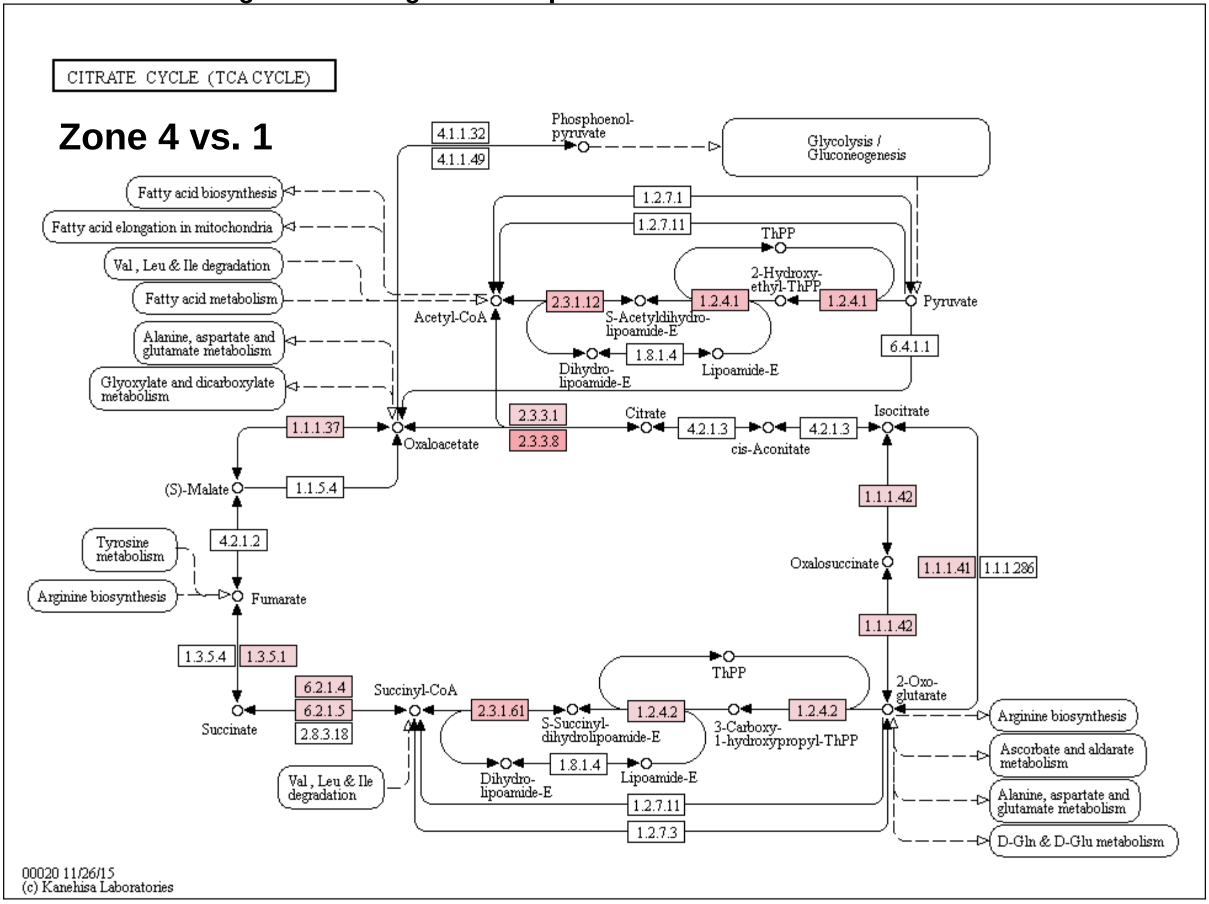

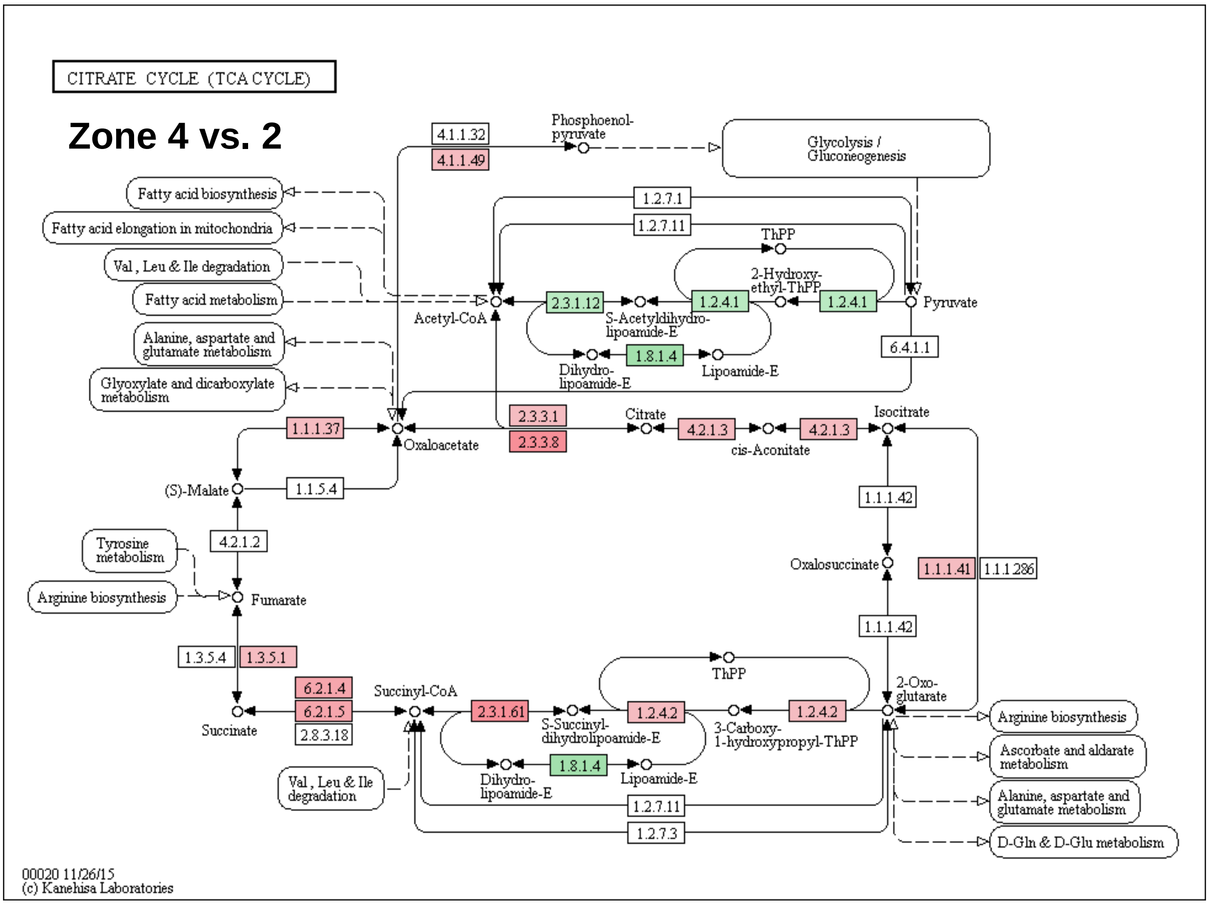

KAAS Kegg mapping of *L. sativa* cv. Tizian DEG's

Only DEG's with p-adjusted values of 0.05 or smaller and absolute fold changes of 2 or higher are depicted

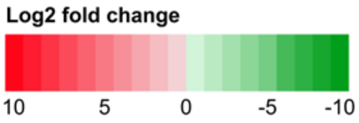

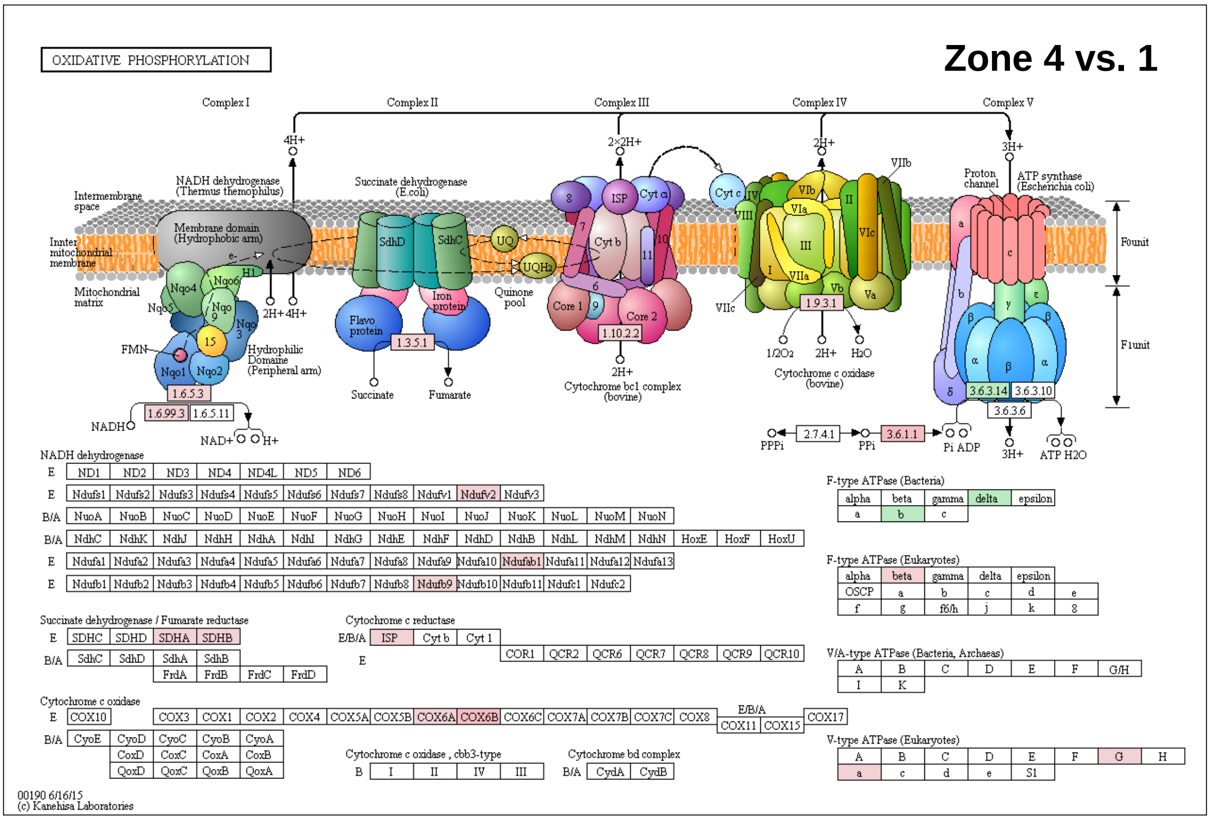

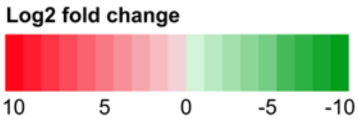

KAAS Kegg mapping of *L. sativa* cv. Tizian DEG's

Only DEG's with p-adjusted values of 0.05 or smaller and absolute fold changes of 2 or higher are depicted

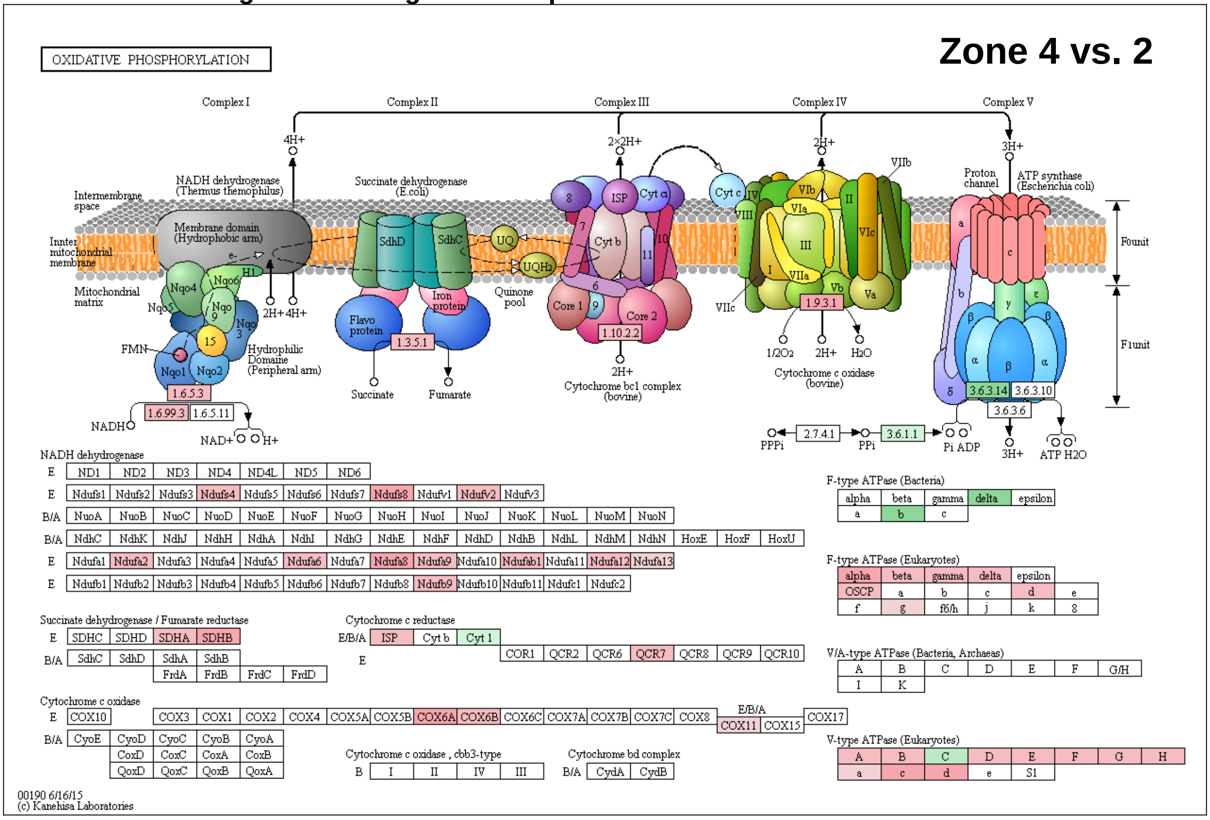

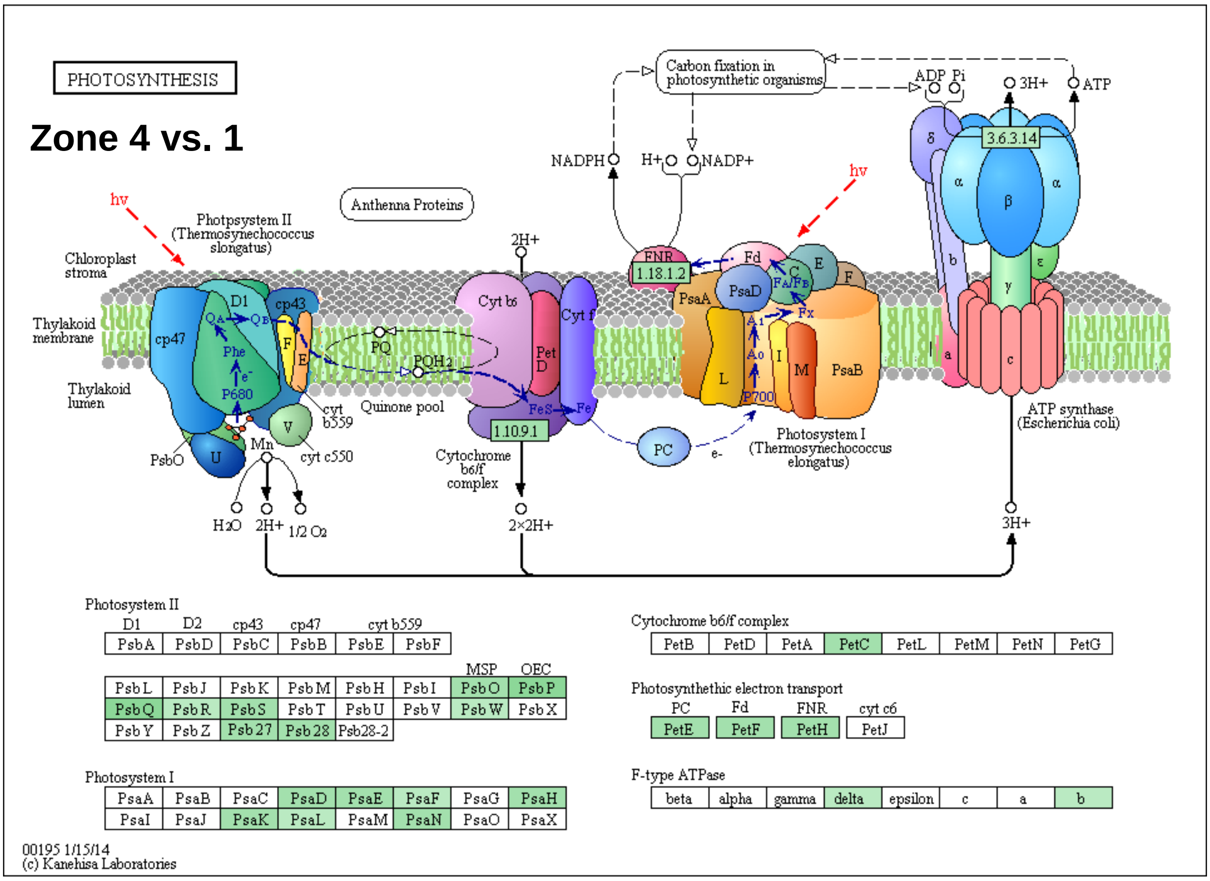

KAAS Kegg mapping of *L. sativa* cv. Tizian DEG's

Only DEG's with p-adjusted values of 0.05 or smaller and absolute fold changes of 2 or higher are depicted

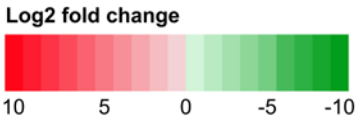

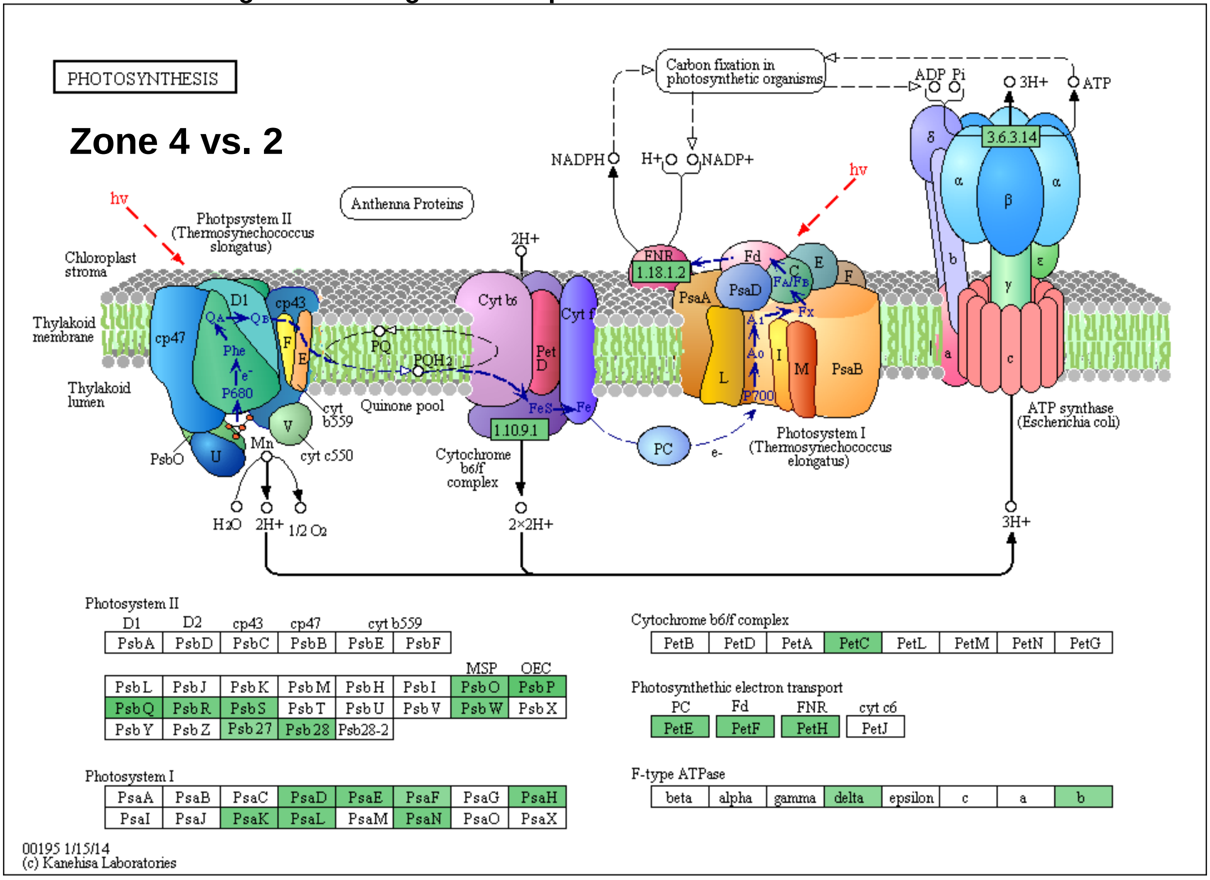

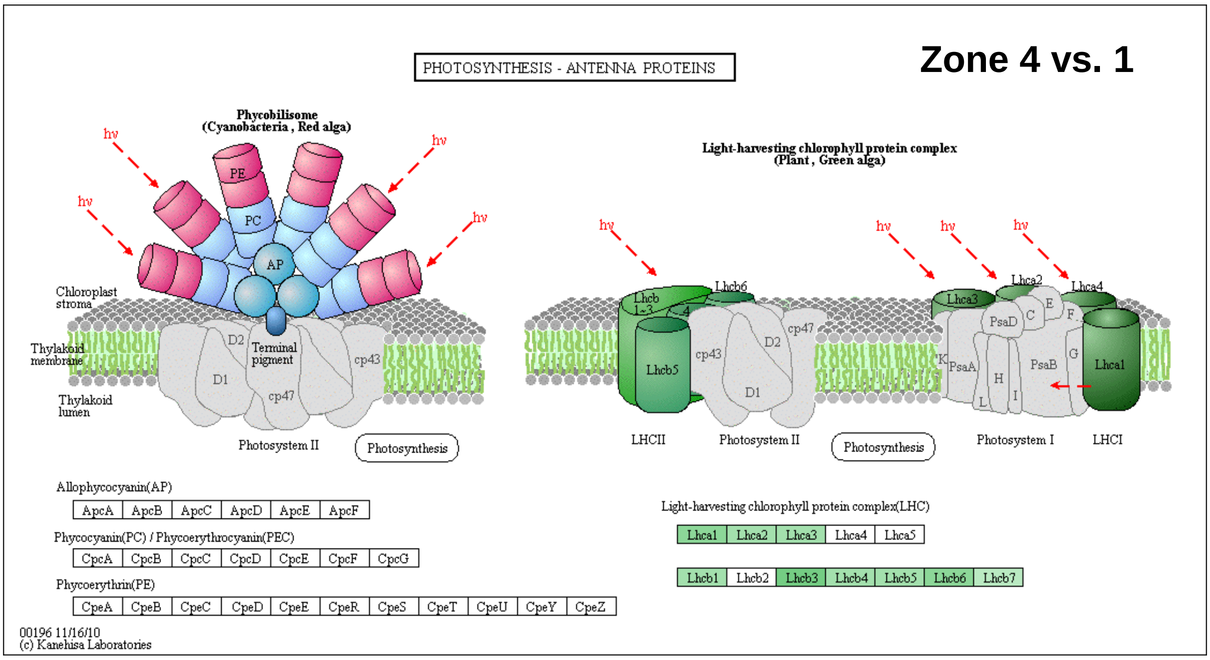

KAAS Kegg mapping of *L. sativa* cv. Tizian DEG’s

Only DEG’s with p-adjusted values of 0.05 or smaller and absolute fold changes of 2 or higher are depicted

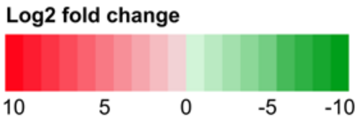

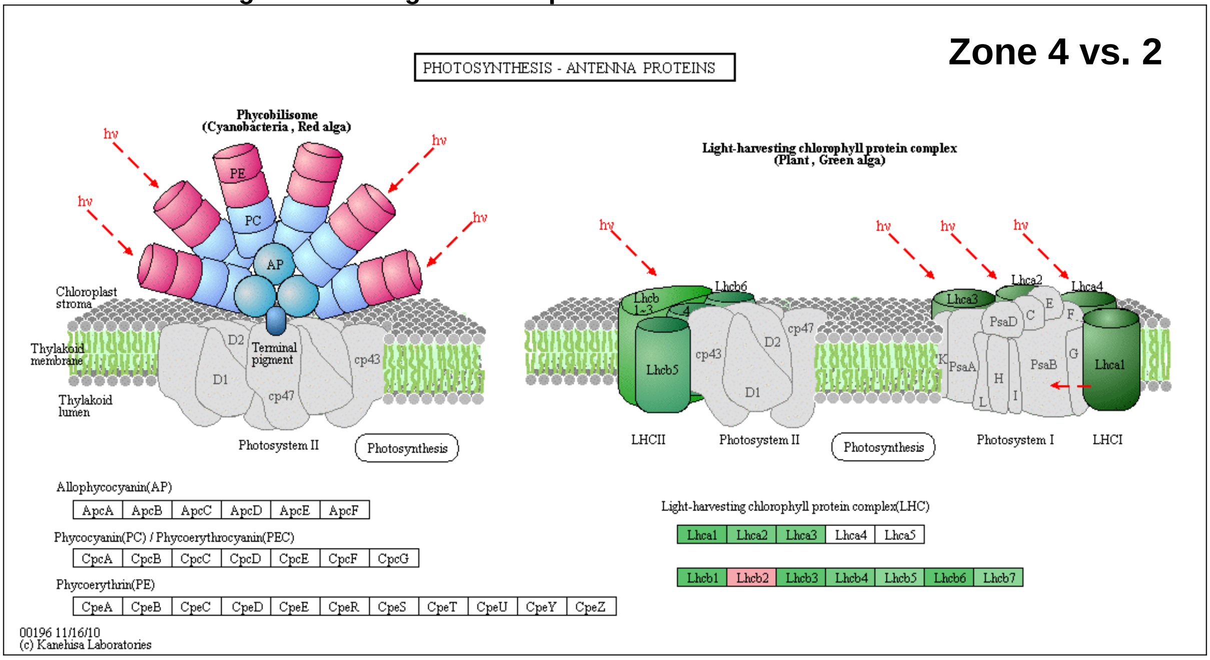

KAAS Kegg mapping of *L. sativa* cv. Tizian DEG's

Only DEG's with p-adjusted values of 0.05 or smaller and absolute fold changes of 2 or higher are depicted

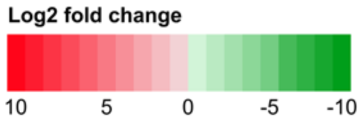

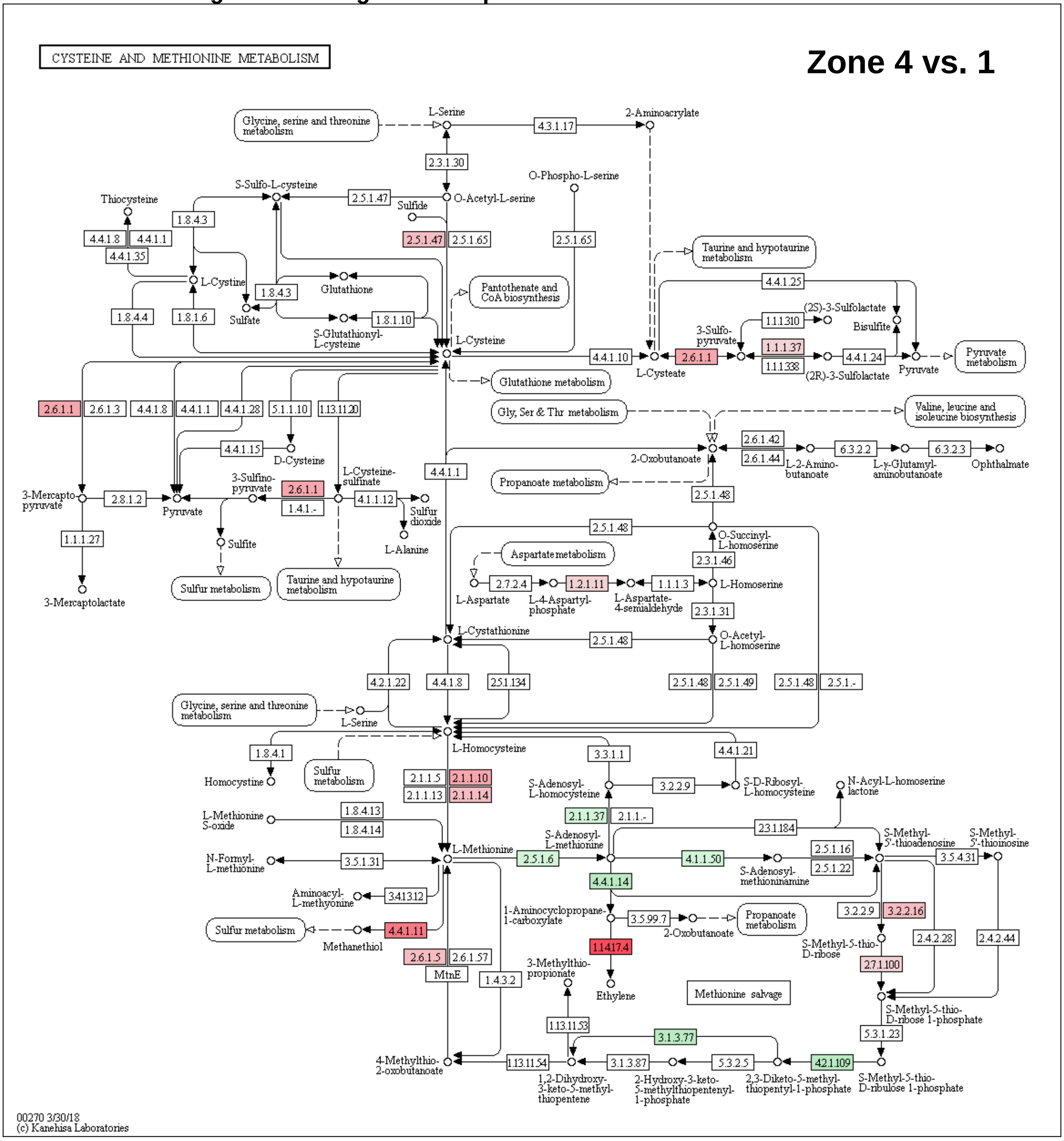

KAAS Kegg mapping of *L. sativa* cv. Tizian DEG's

Only DEG's with p-adjusted values of 0.05 or smaller and absolute fold changes of 2 or higher are depicted

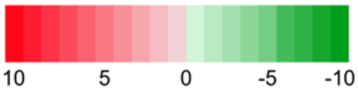

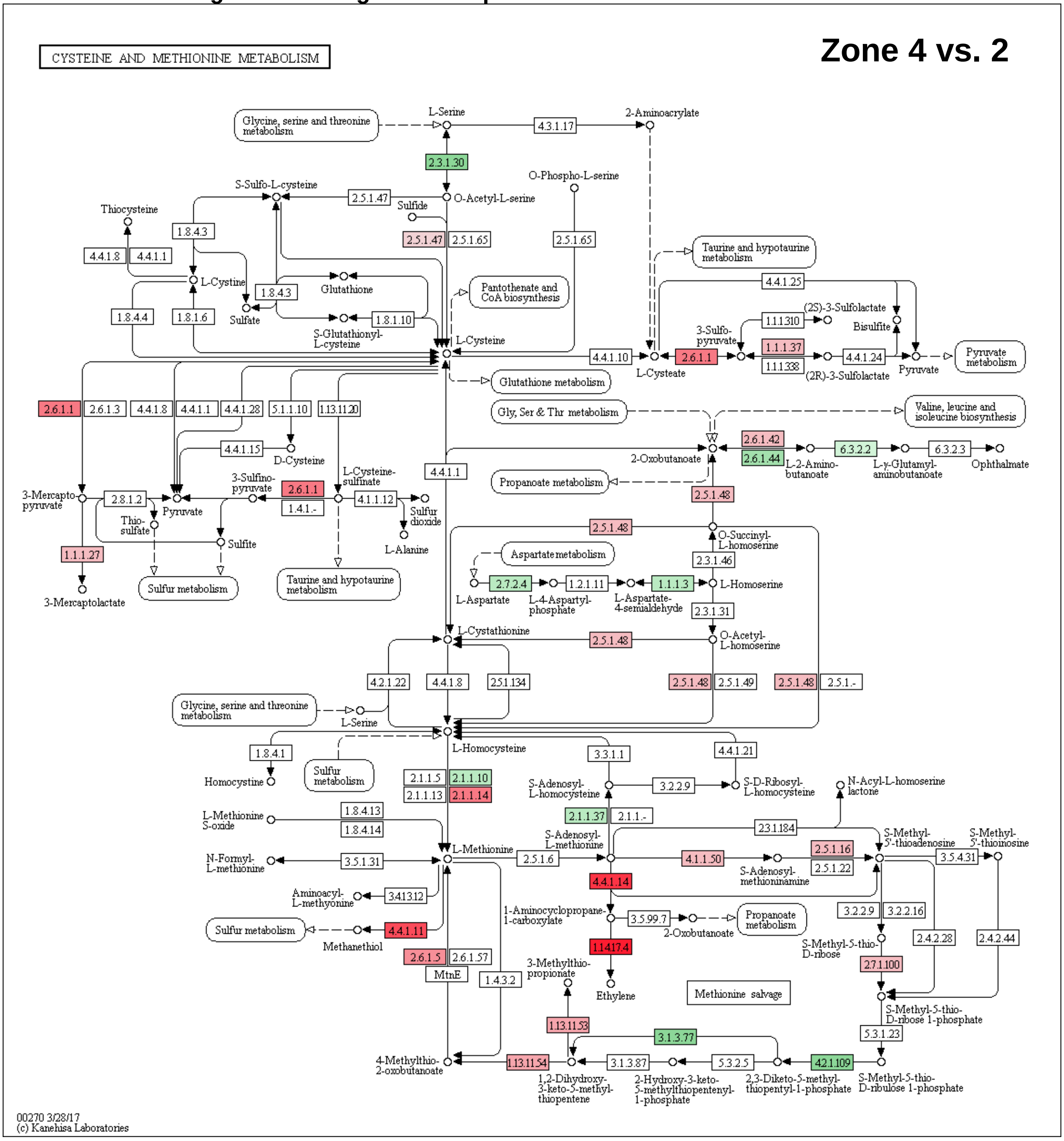

KAAS Kegg mapping of *L. sativa* cv. Tizian DEG’s

Only DEG’s with p-adjusted values of 0.05 or smaller and absolute fold changes of 2 or higher are depicted

Log2 fold change

10 5 0 -5 -10

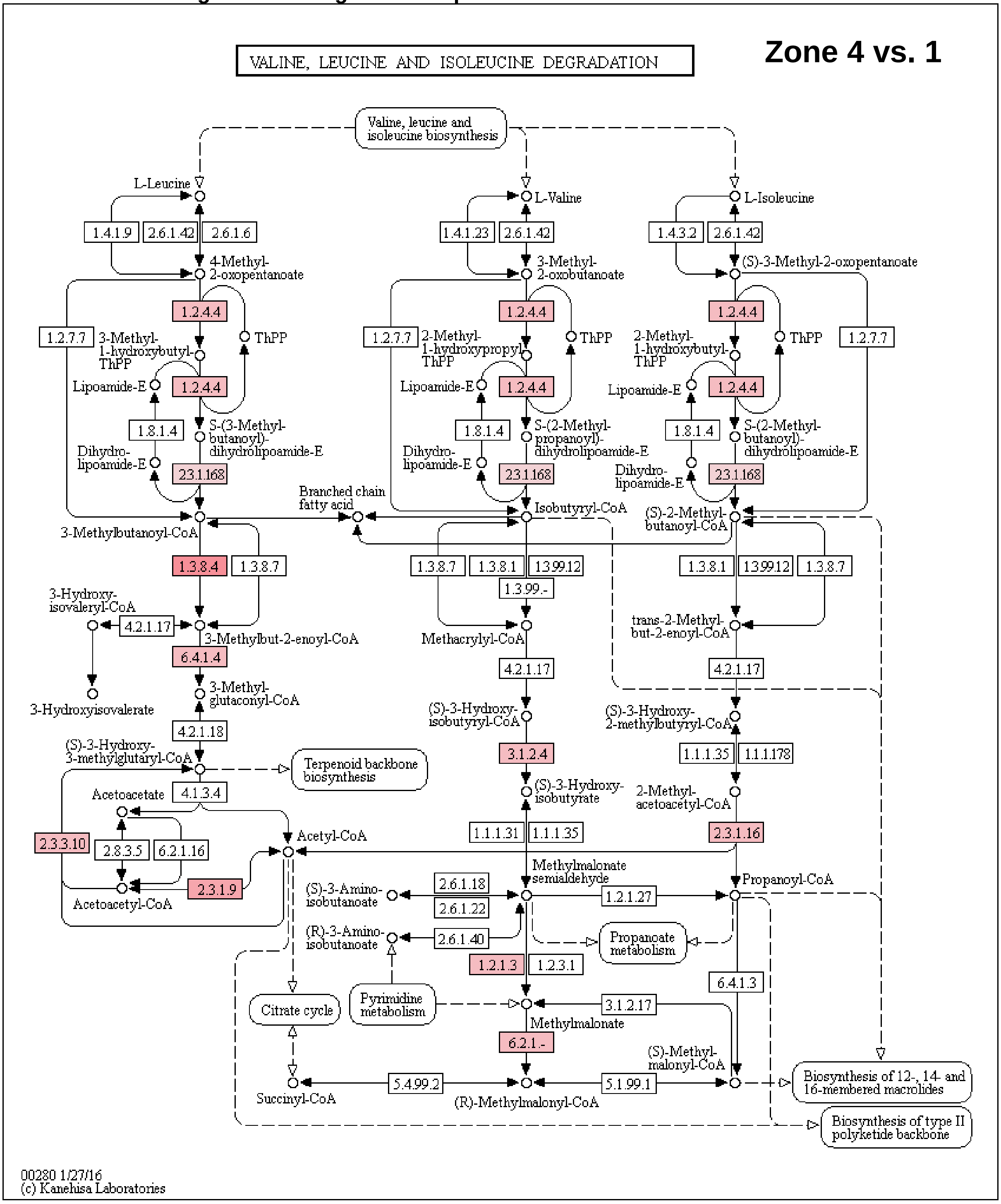

KAAS Kegg mapping of *L. sativa* cv. Tizian DEG's

Only DEG's with p-adjusted values of 0.05 or smaller and absolute fold changes of 2 or higher are depicted

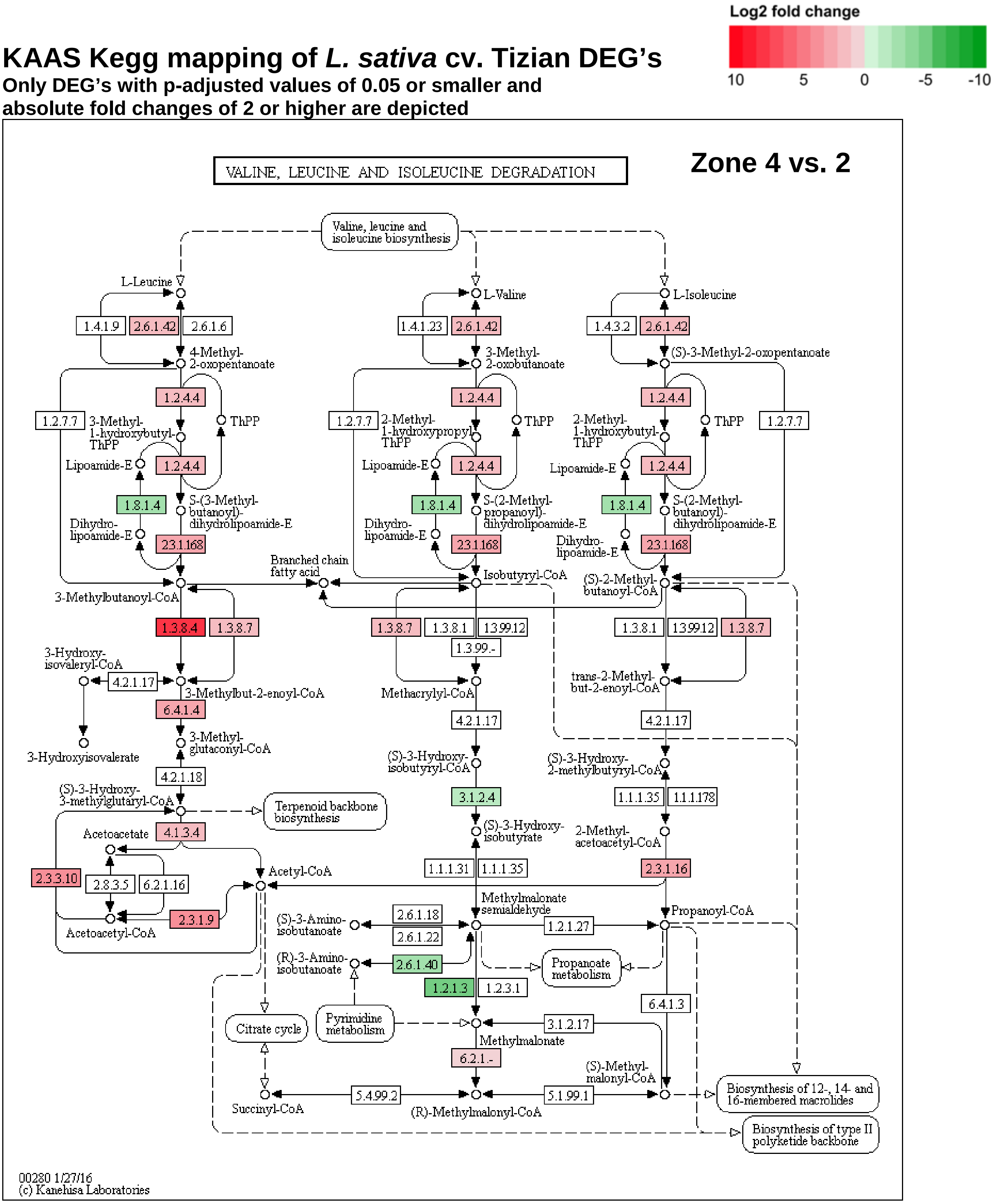

KAAS Kegg mapping of *L. sativa* cv. Tizian DEG's

Only DEG's with p-adjusted values of 0.05 or smaller and absolute fold changes of 2 or higher are depicted

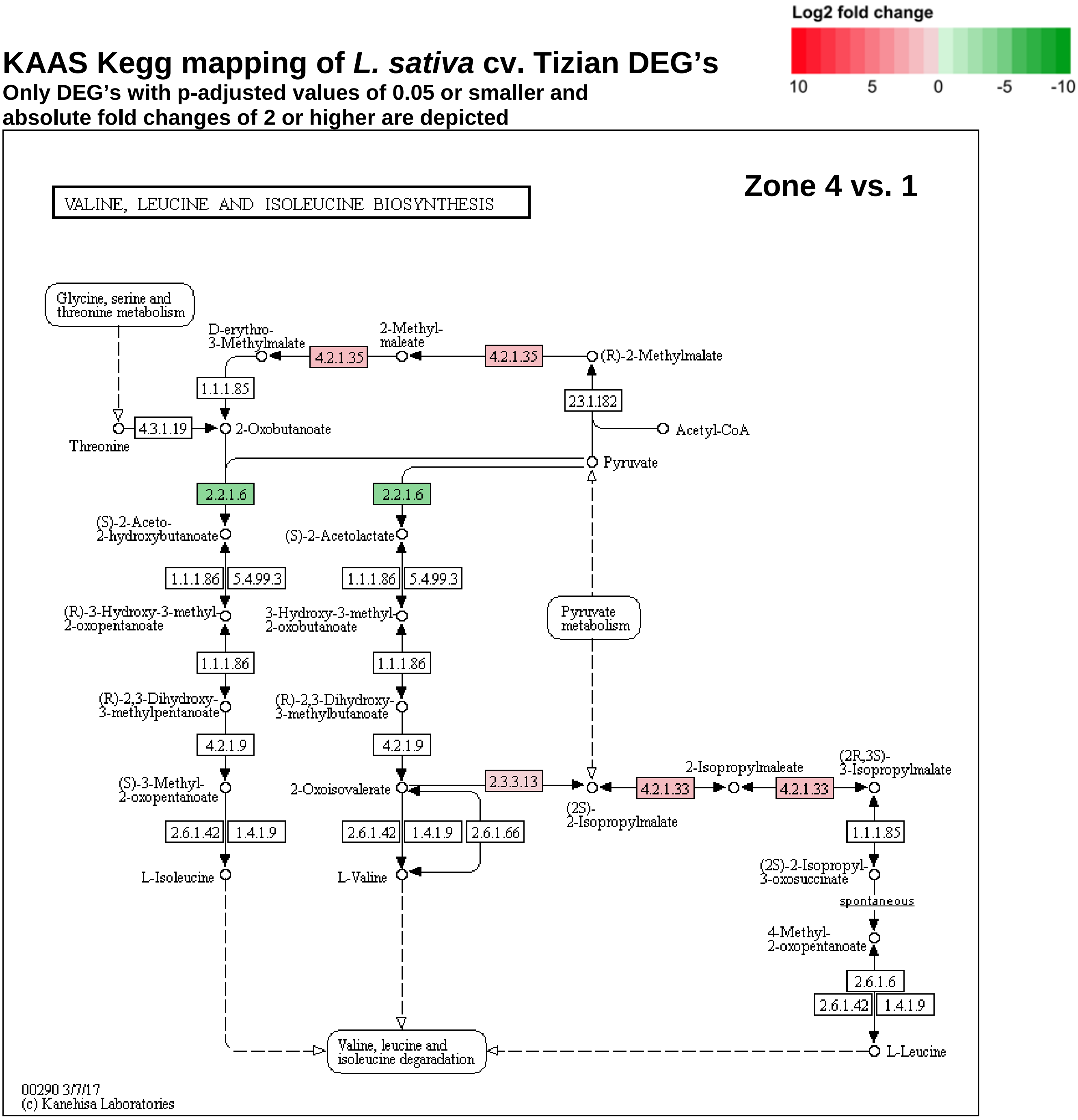

KAAS Kegg mapping of *L. sativa* cv. Tizian DEG's

Only DEG's with p-adjusted values of 0.05 or smaller and absolute fold changes of 2 or higher are depicted

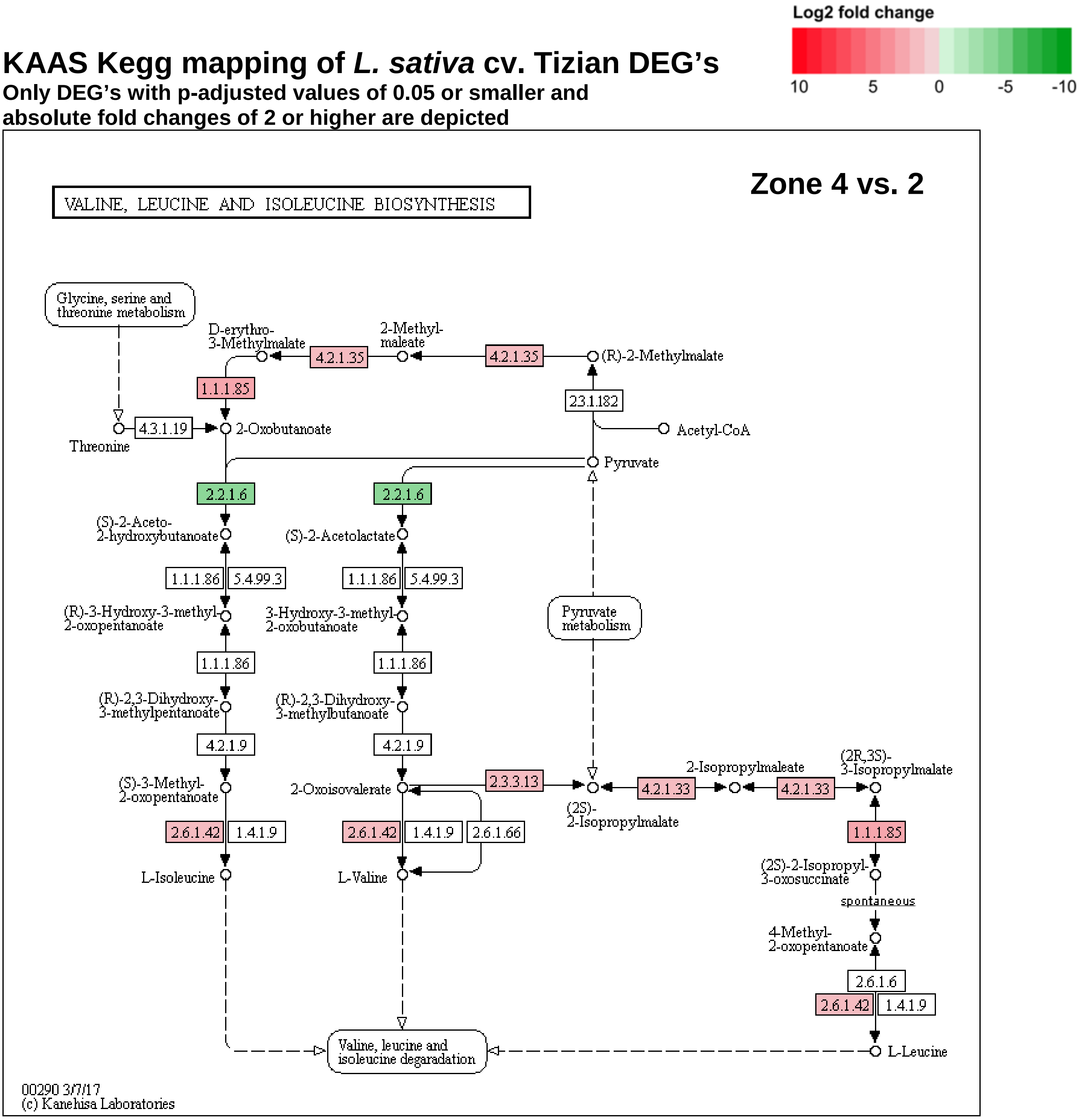

KAAS Kegg mapping of *L. sativa* cv. Tizian DEG's

Only DEG's with p-adjusted values of 0.05 or smaller and absolute fold changes of 2 or higher are depicted

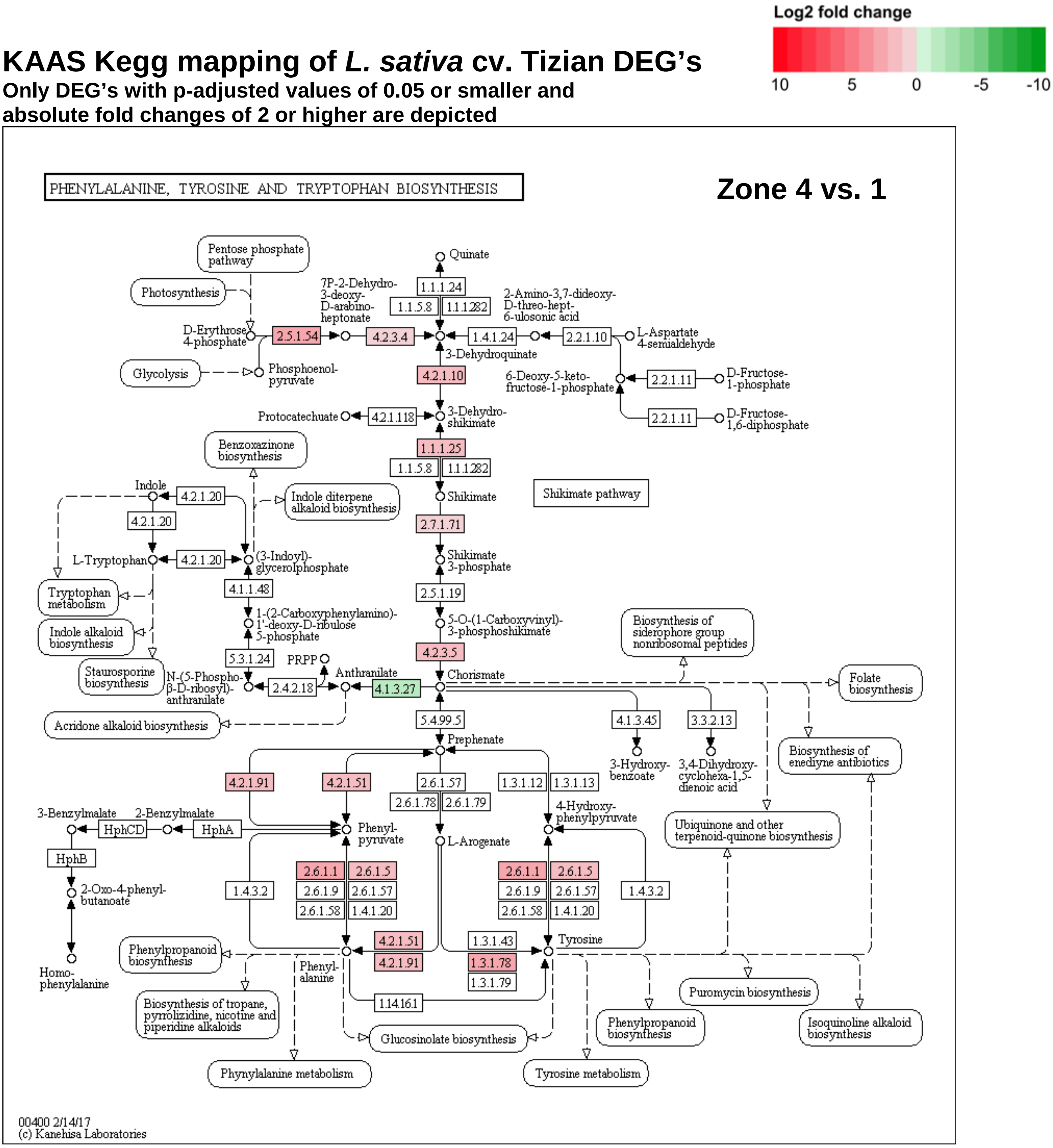

KAAS Kegg mapping of *L. sativa* cv. Tizian DEG's

Only DEG's with p-adjusted values of 0.05 or smaller and absolute fold changes of 2 or higher are depicted

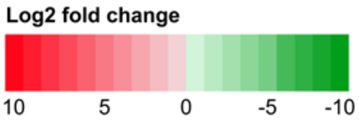

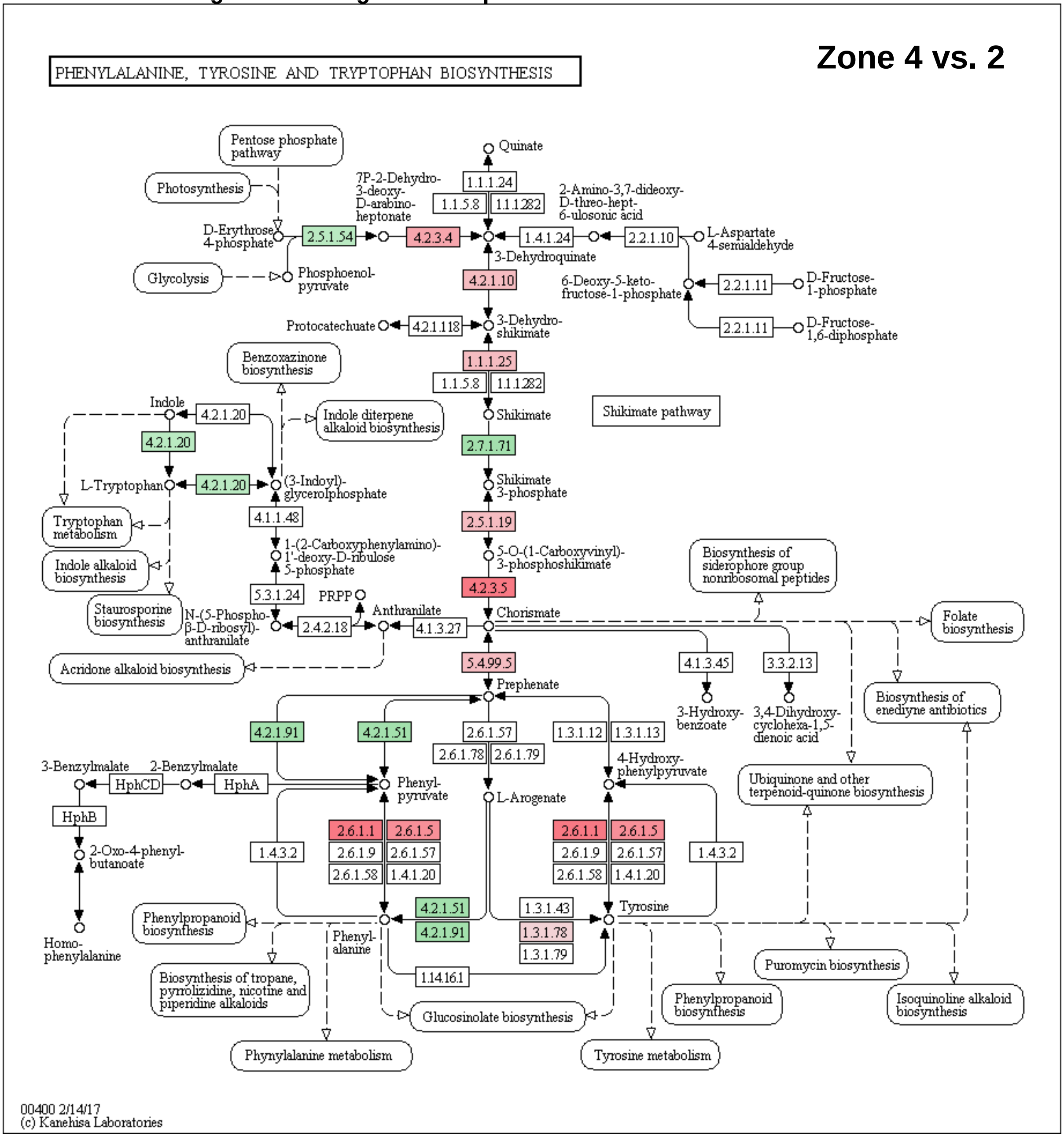

KAAS Kegg mapping of *L. sativa* cv. Tizian DEG's

Only DEG's with p-adjusted values of 0.05 or smaller and absolute fold changes of 2 or higher are depicted

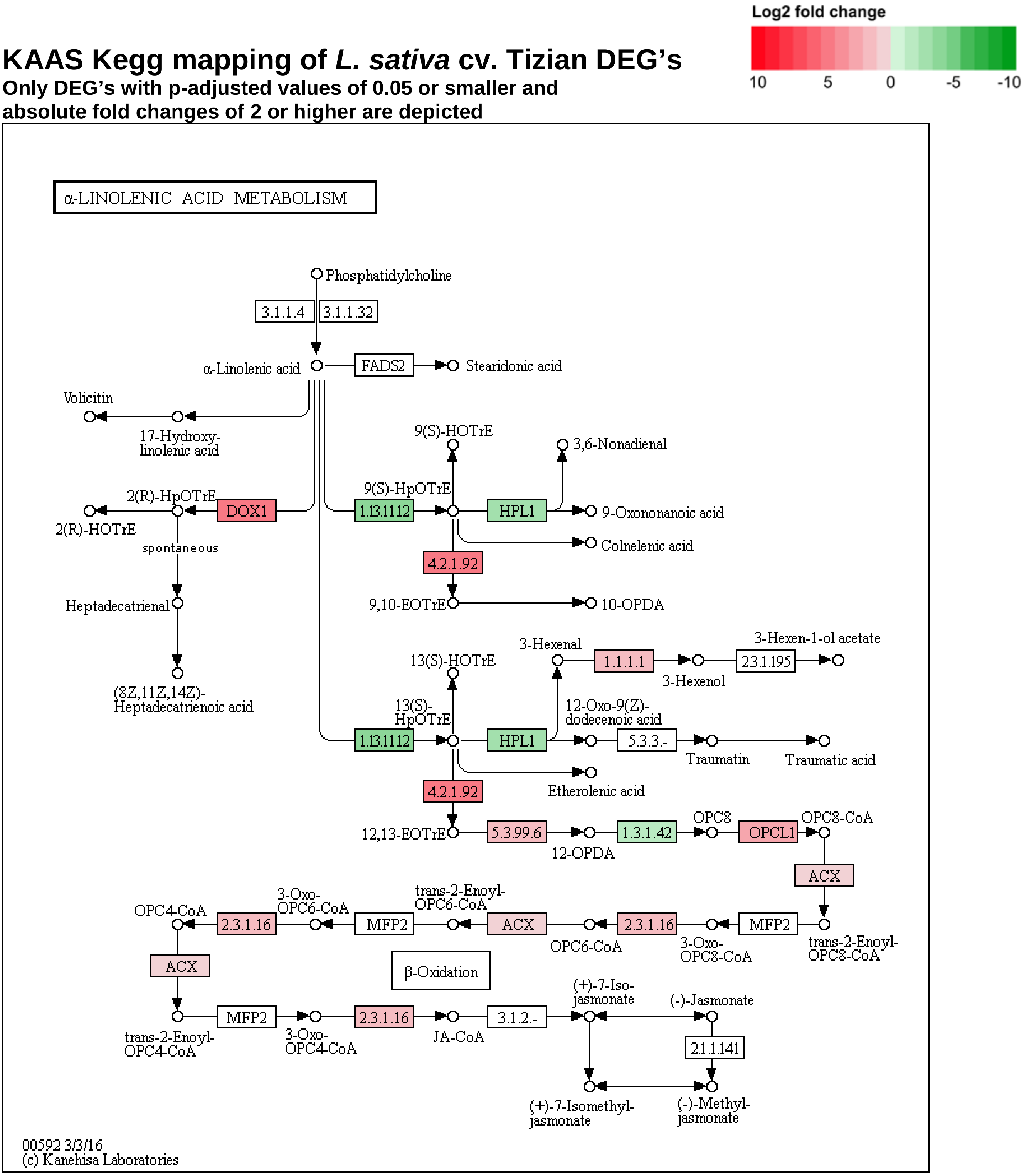

KAAS Kegg mapping of *L. sativa* cv. Tizian DEG's

Only DEG's with p-adjusted values of 0.05 or smaller and absolute fold changes of 2 or higher are depicted

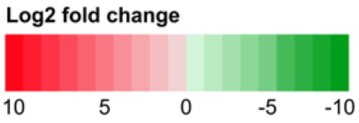

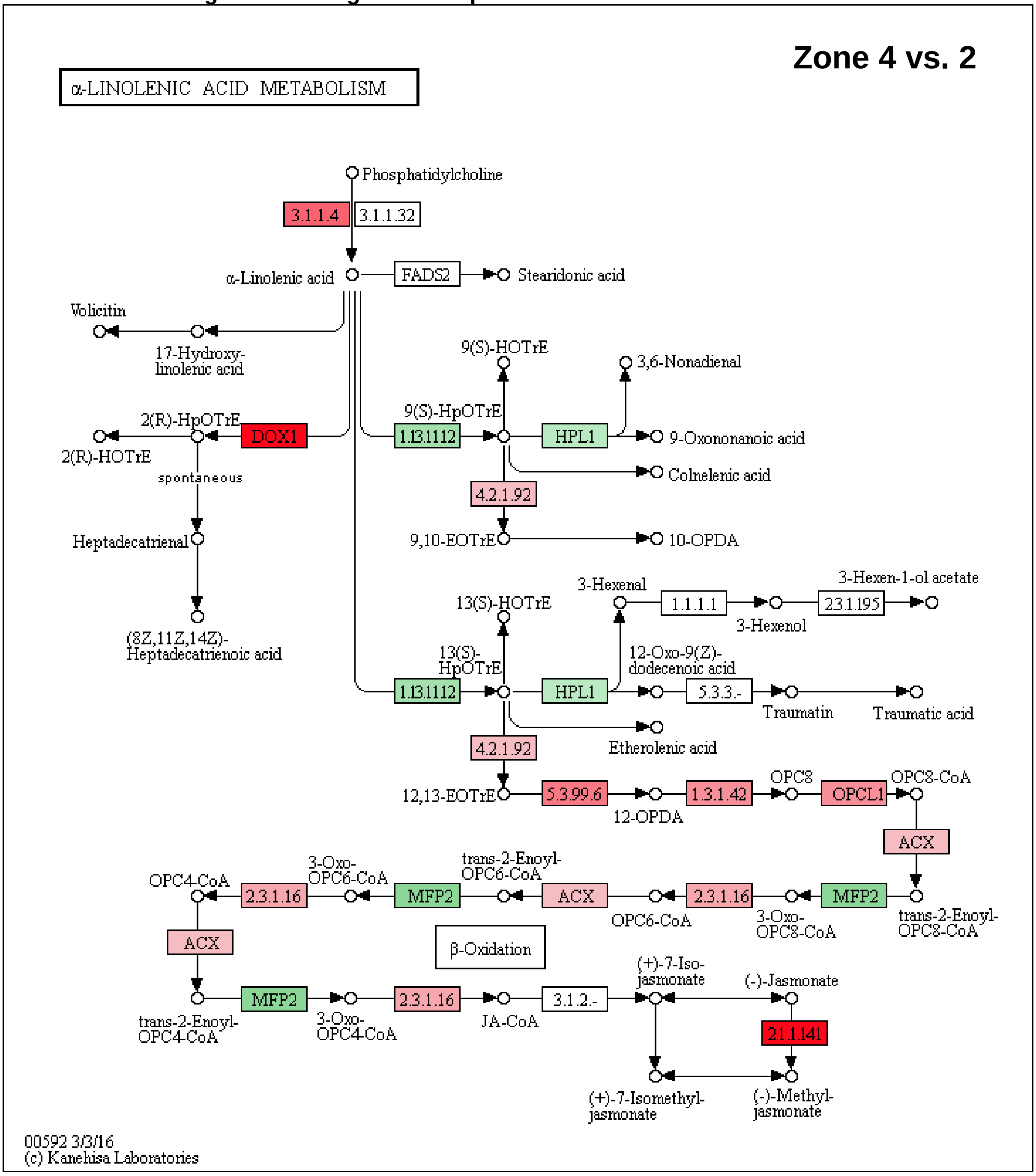

KAAS Kegg mapping of *L. sativa* cv. Tizian DEG's

Only DEG's with p-adjusted values of 0.05 or smaller and absolute fold changes of 2 or higher are depicted

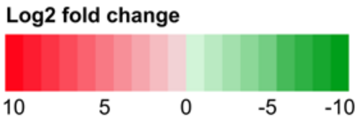

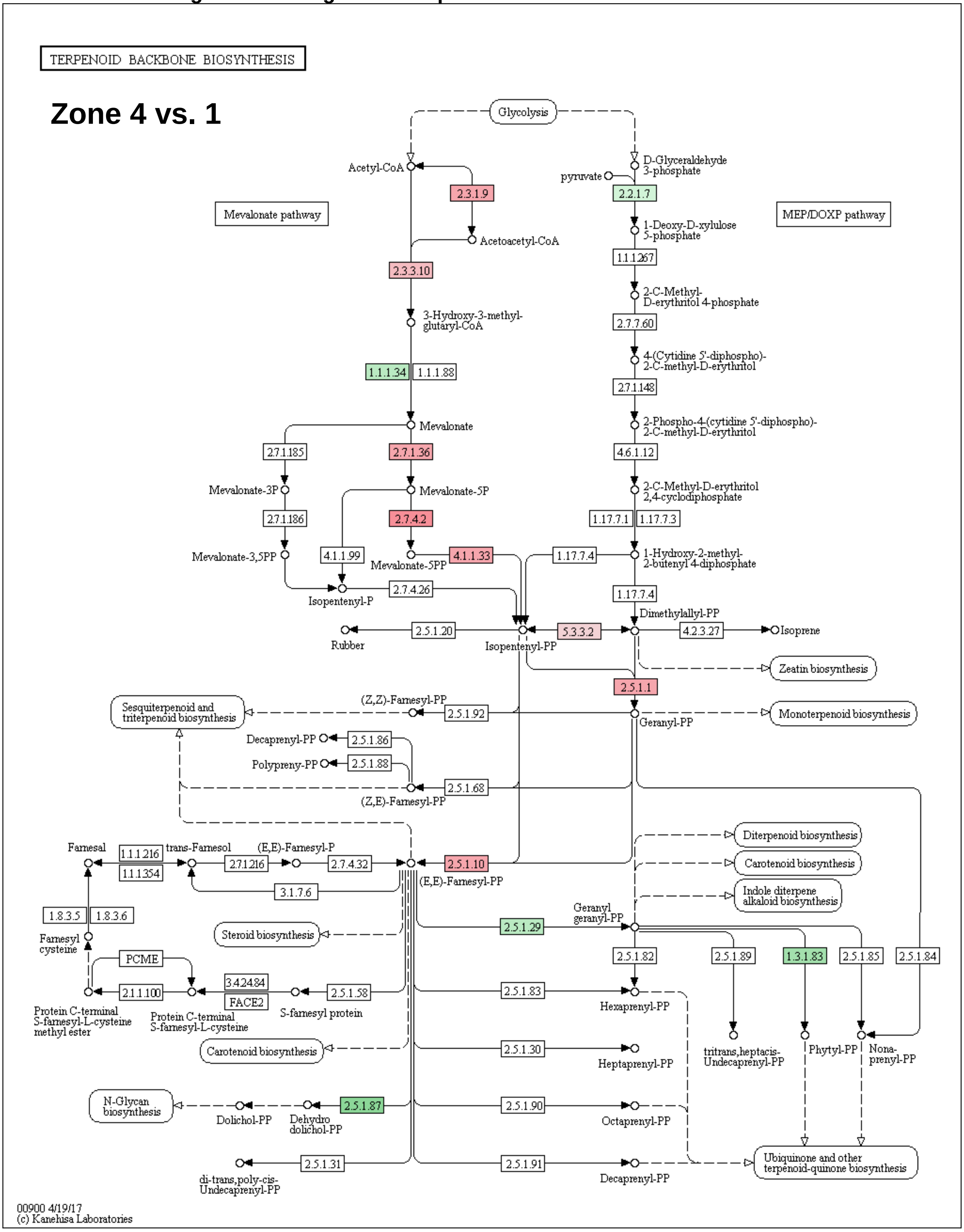

KAAS Kegg mapping of *L. sativa* cv. Tizian DEG's

Only DEG's with p-adjusted values of 0.05 or smaller and absolute fold changes of 2 or higher are depicted

Log2 fold change

10 5 0 -5 -10

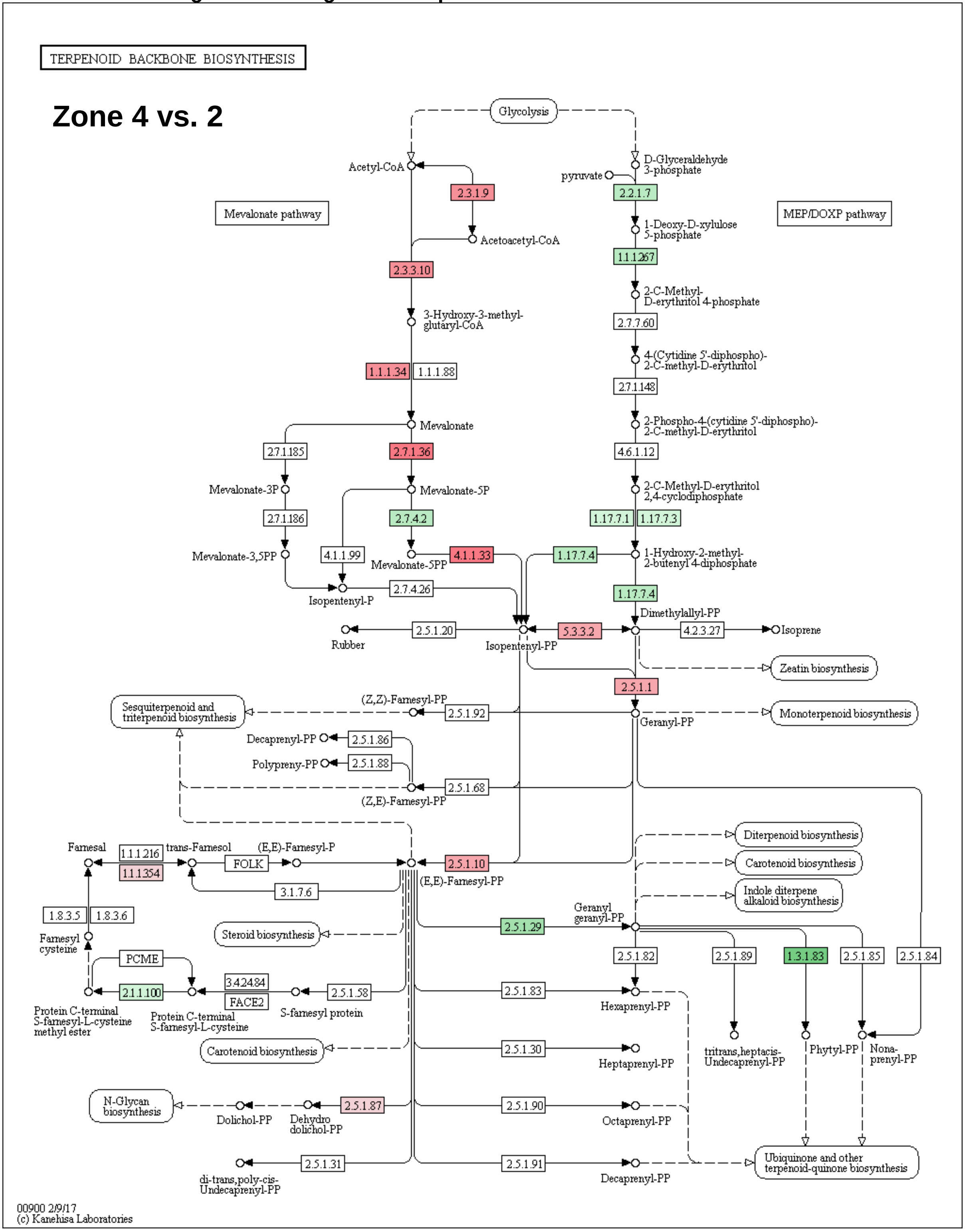

KAAS Kegg mapping of *L. sativa* cv. Tizian DEG’s

Only DEG’s with p-adjusted values of 0.05 or smaller and absolute fold changes of 2 or higher are depicted

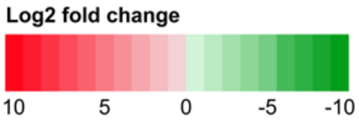

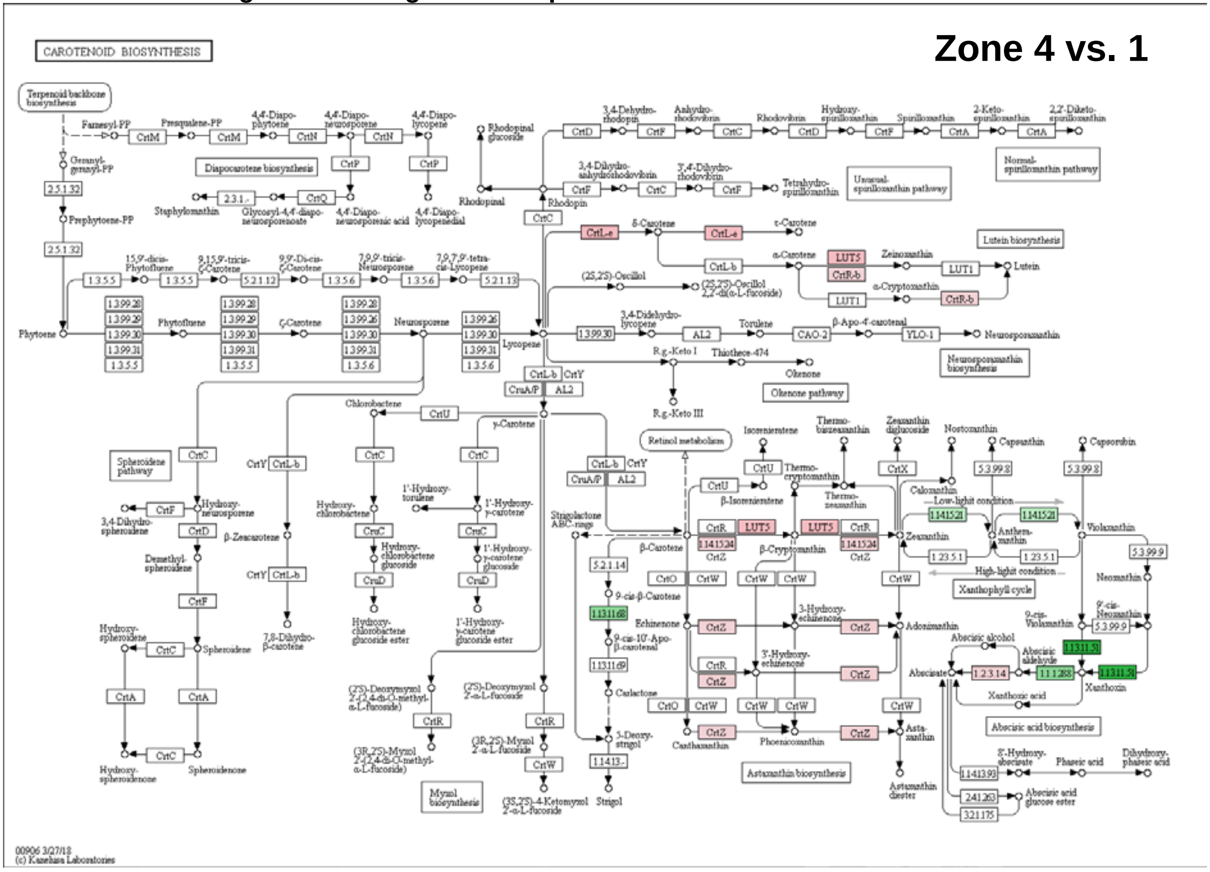

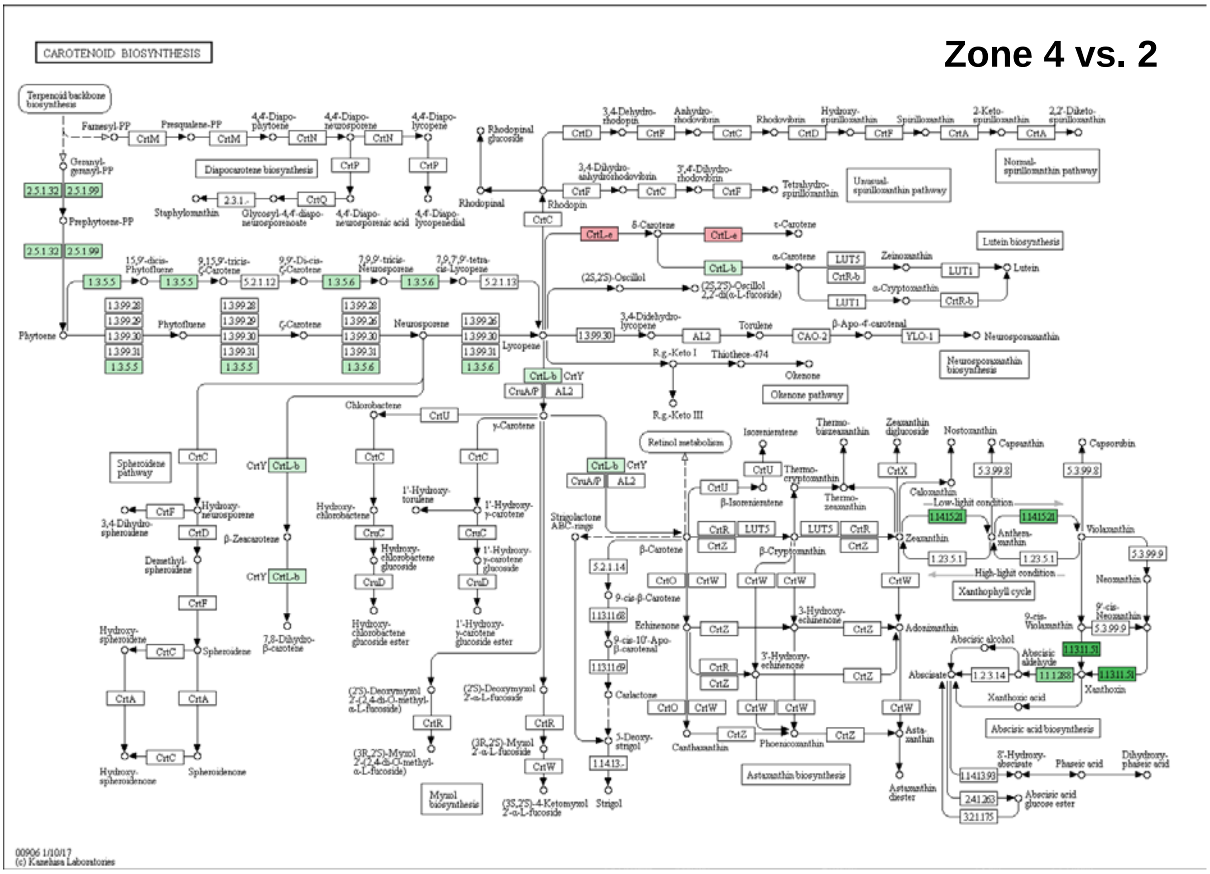

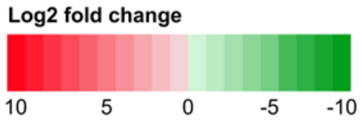

KAAS Kegg mapping of *L. sativa* cv. Tizian DEG's

Only DEG's with p-adjusted values of 0.05 or smaller and absolute fold changes of 2 or higher are depicted

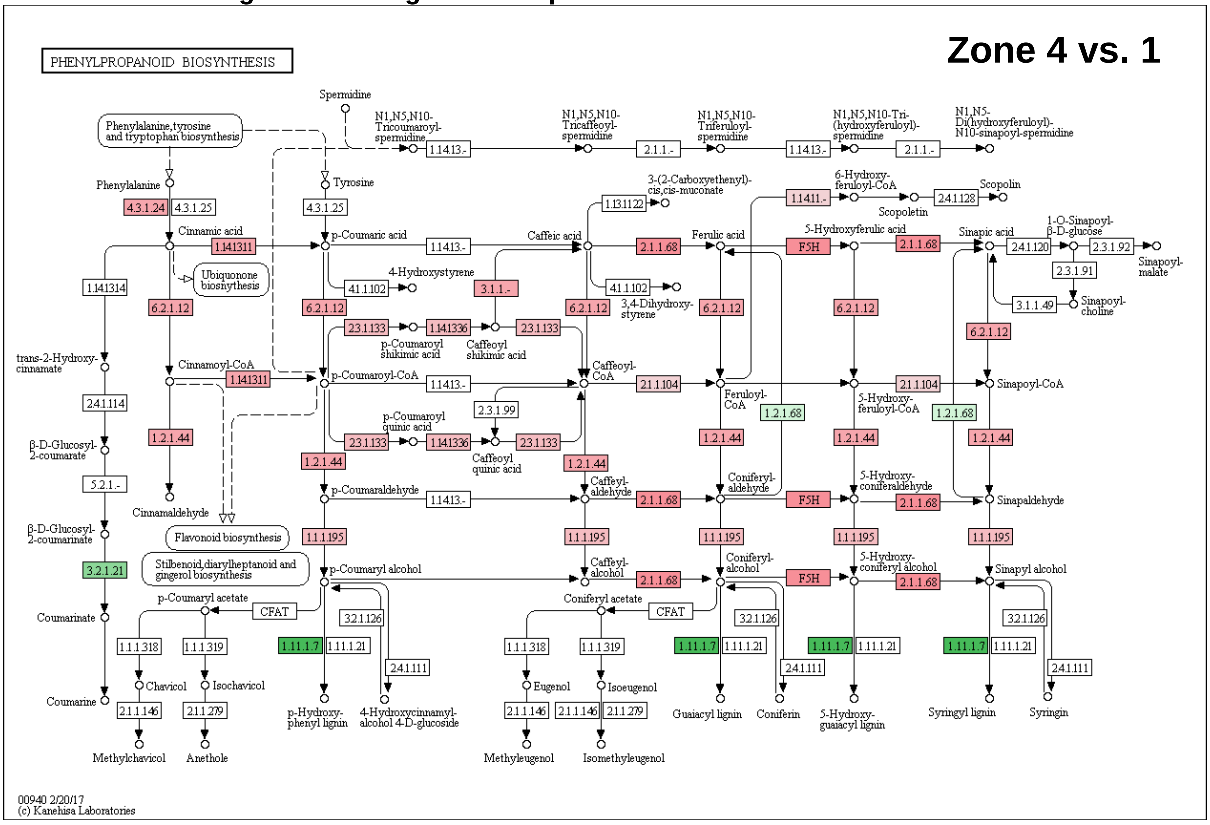

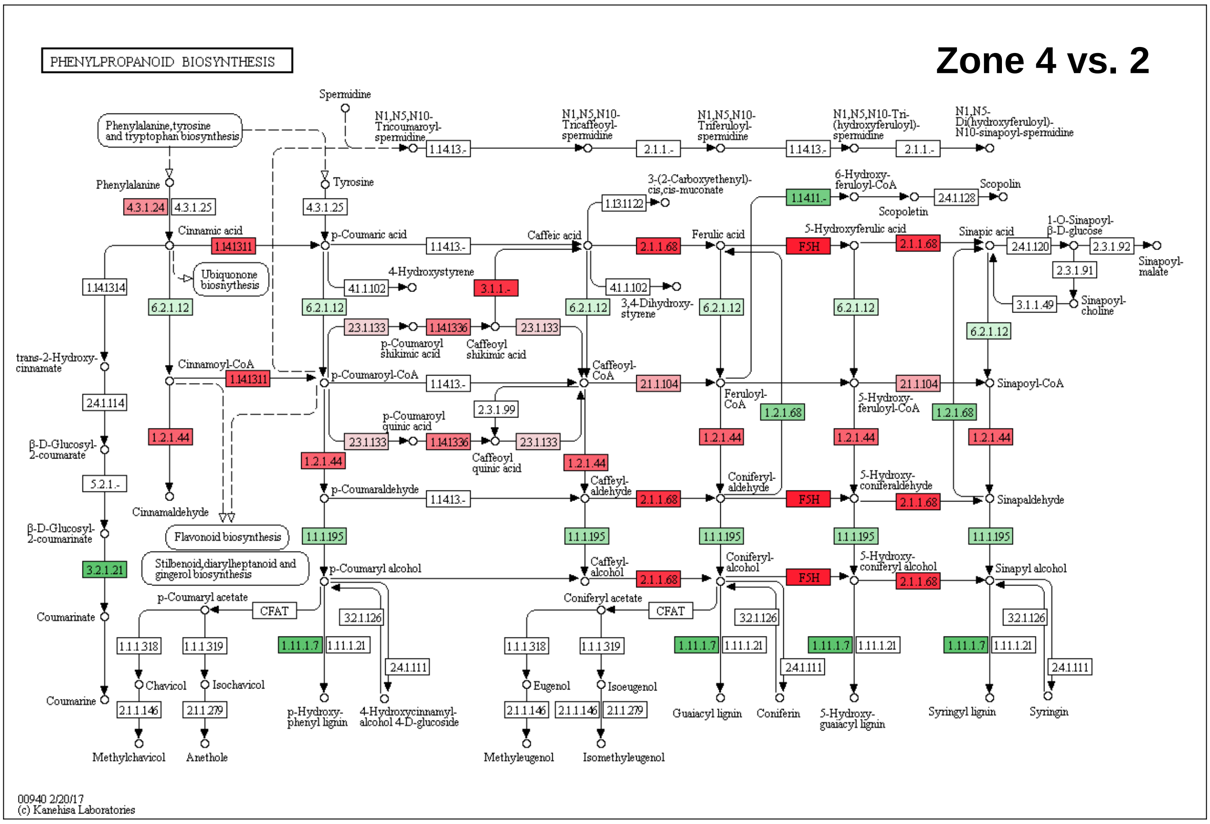

KAAS Kegg mapping of *L. sativa* cv. Tizian DEG's

Only DEG's with p-adjusted values of 0.05 or smaller and absolute fold changes of 2 or higher are depicted

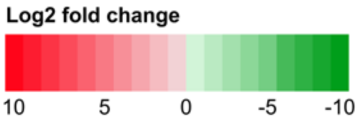

Zone 4 vs. 1

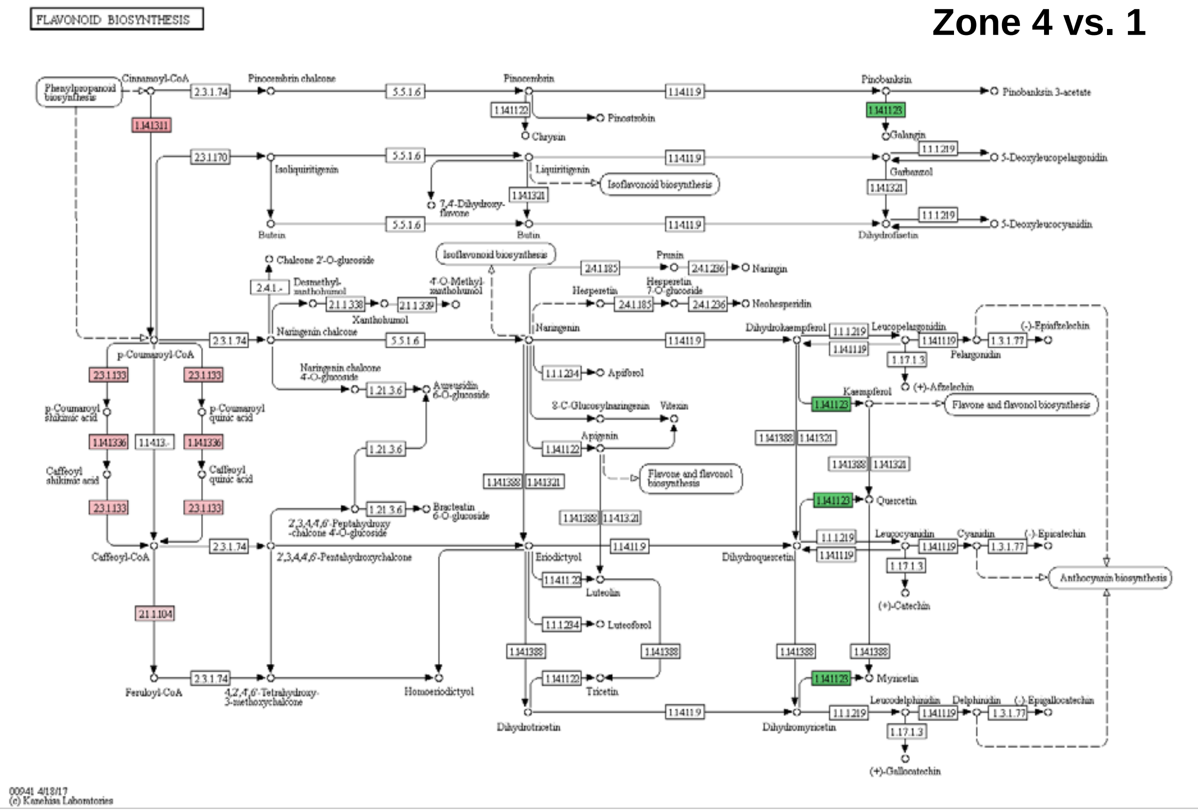

Zone 4 vs. 2

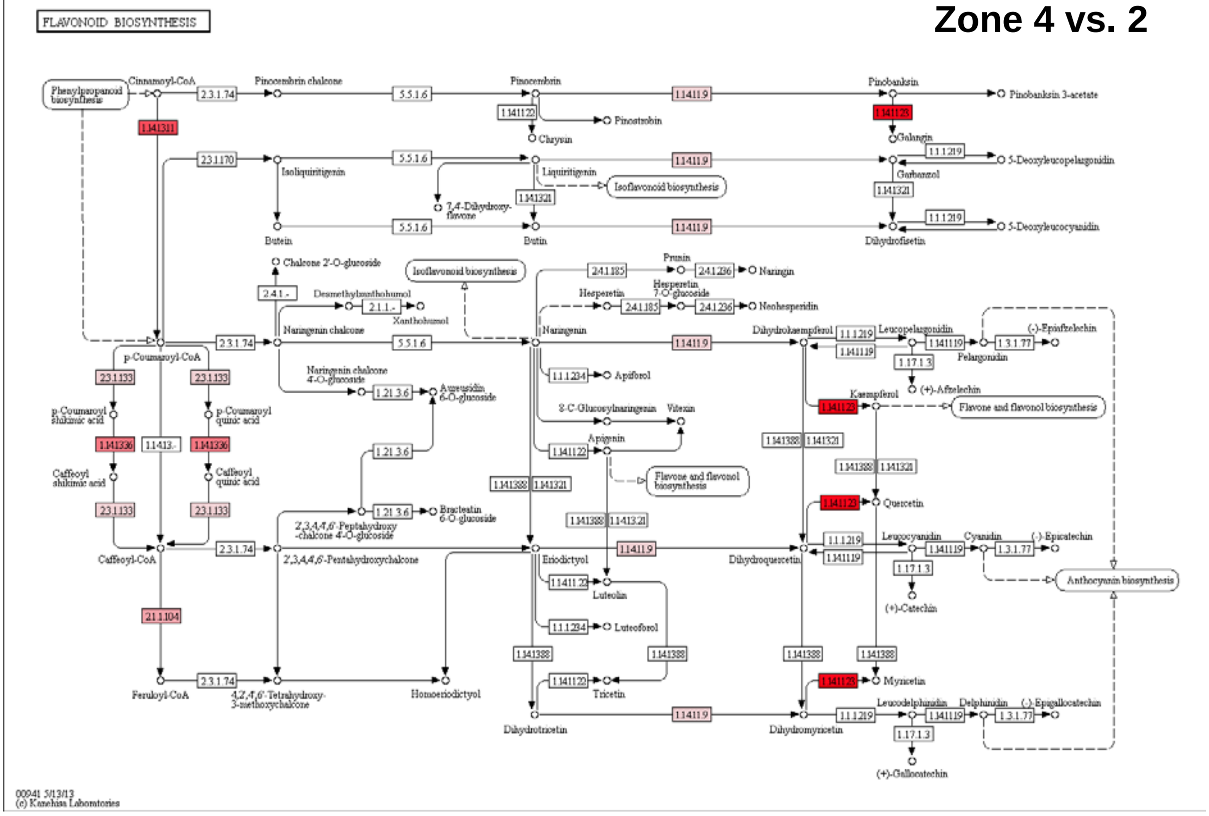

KAAS Kegg mapping of *L. sativa* cv. Tizian DEG's

Only DEG's with p-adjusted values of 0.05 or smaller and absolute fold changes of 2 or higher are depicted

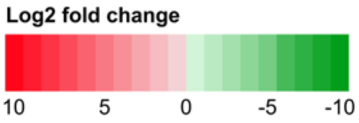

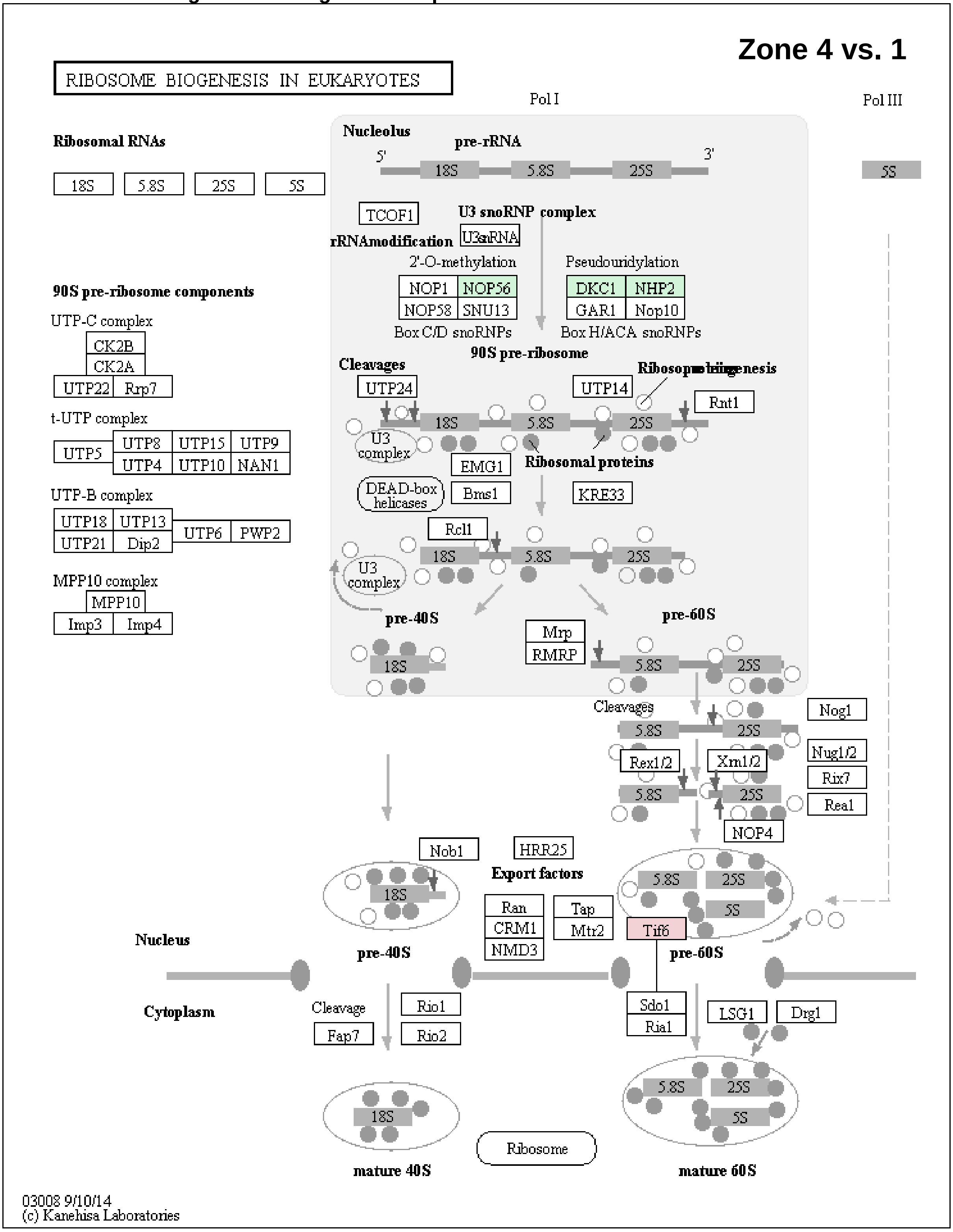

KAAS Kegg mapping of *L. sativa* cv. Tizian DEG's

Only DEG's with p-adjusted values of 0.05 or smaller and absolute fold changes of 2 or higher are depicted

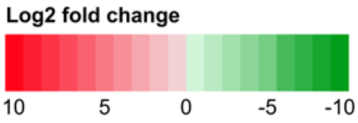

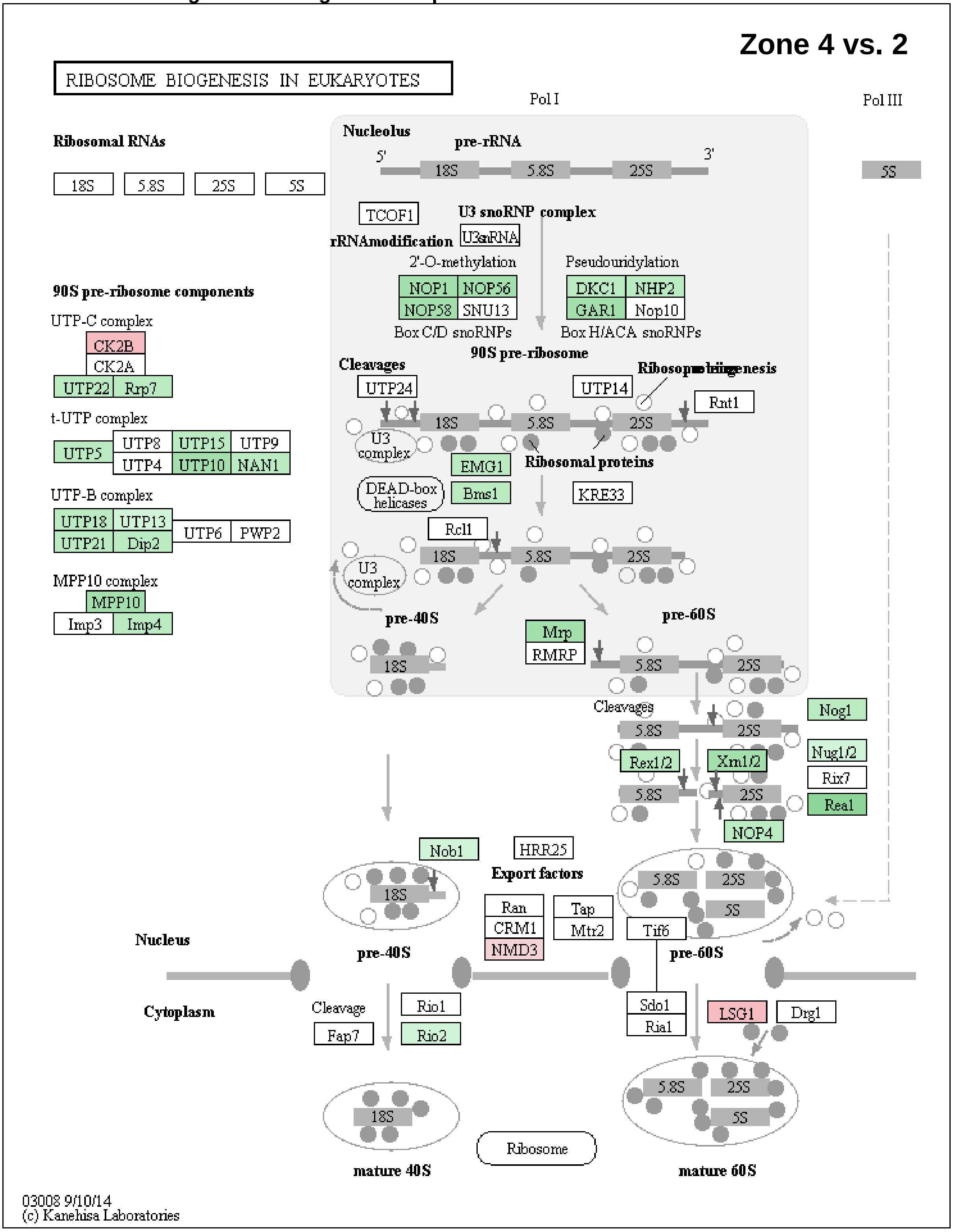

KAAS Kegg mapping of *L. sativa* cv. Tizian DEG’s

Only DEG’s with p-adjusted values of 0.05 or smaller and absolute fold changes of 2 or higher are depicted

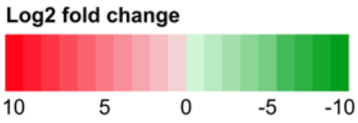

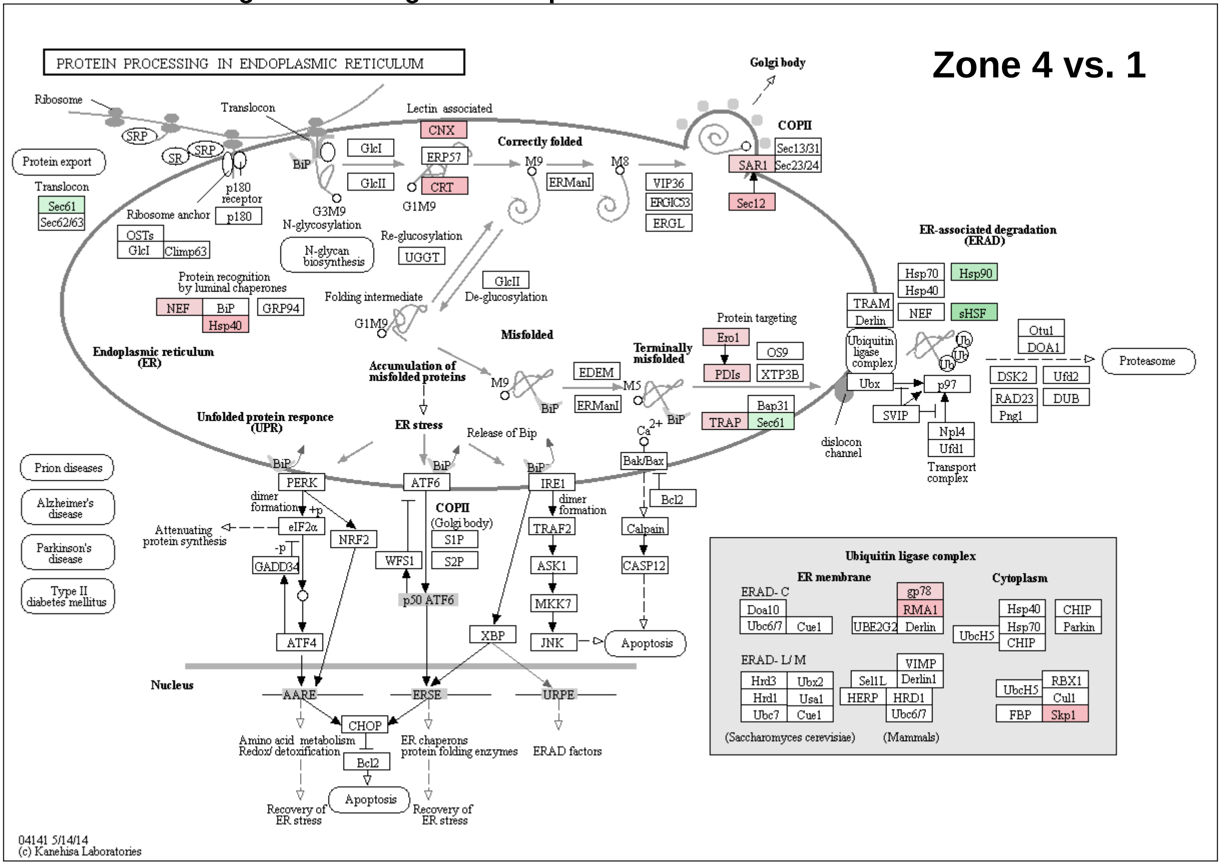

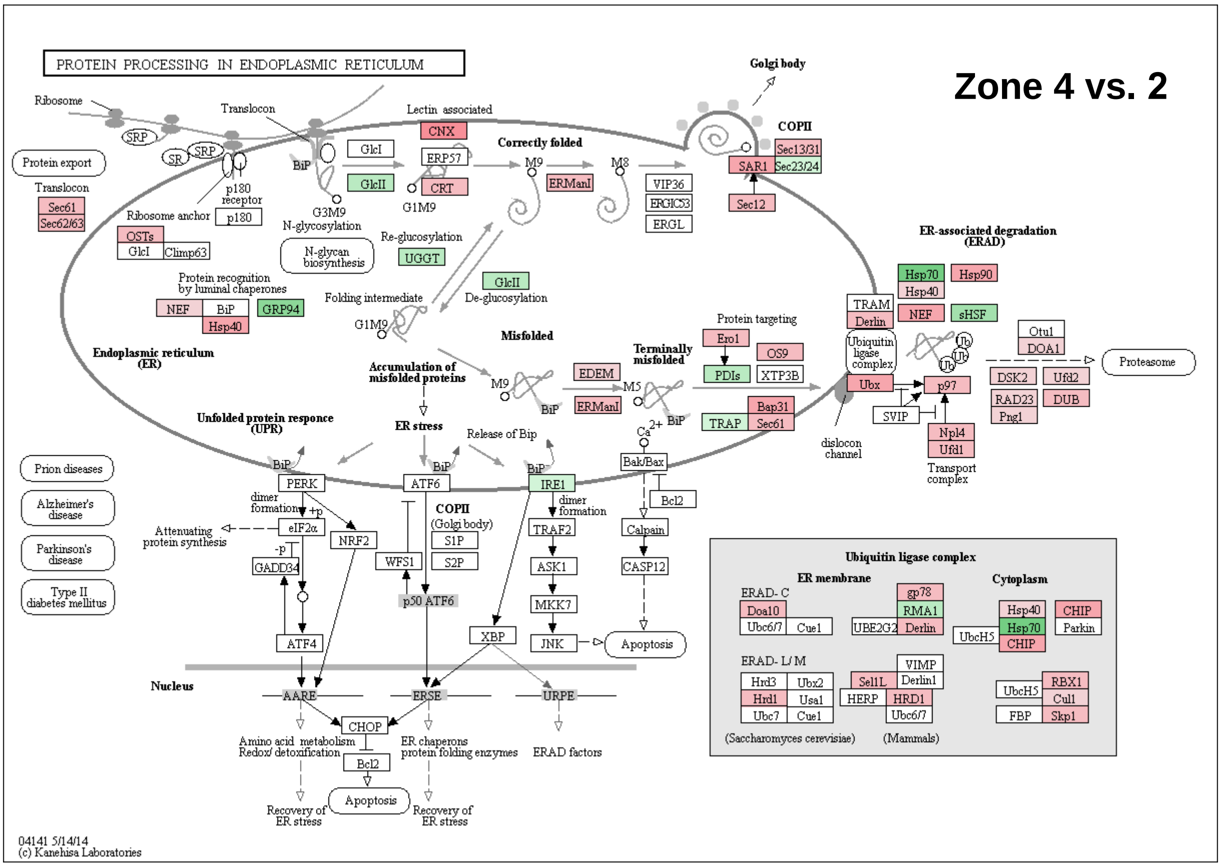

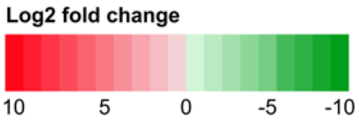

KAAS Kegg mapping of *L. sativa* cv. Tizian DEG's

Only DEG's with p-adjusted values of 0.05 or smaller and absolute fold changes of 2 or higher are depicted

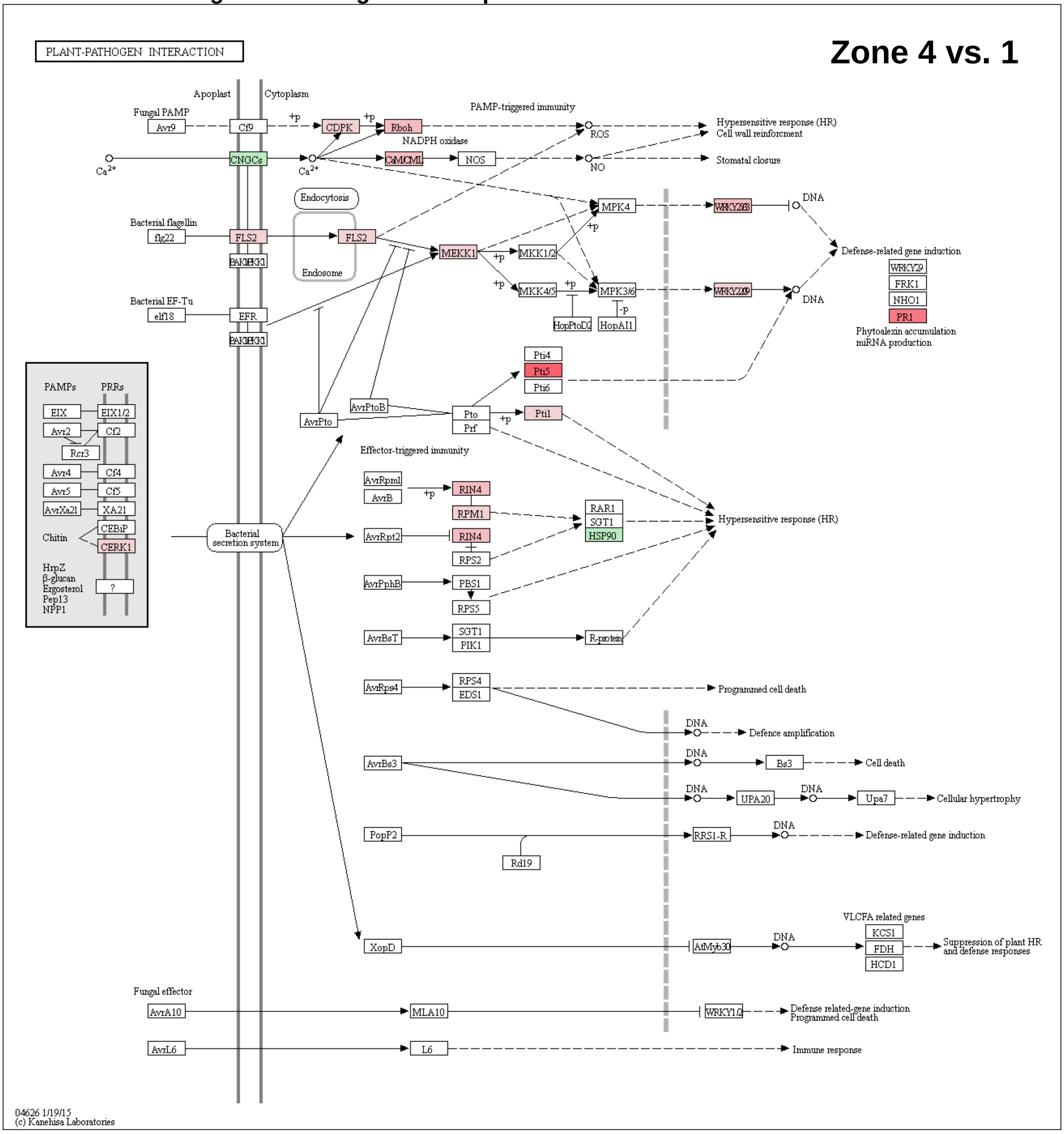

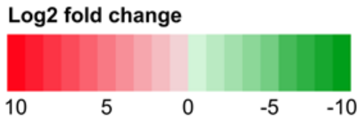

KAAS Kegg mapping of *L. sativa* cv. Tizian DEG's

Only DEG's with p-adjusted values of 0.05 or smaller and absolute fold changes of 2 or higher are depicted

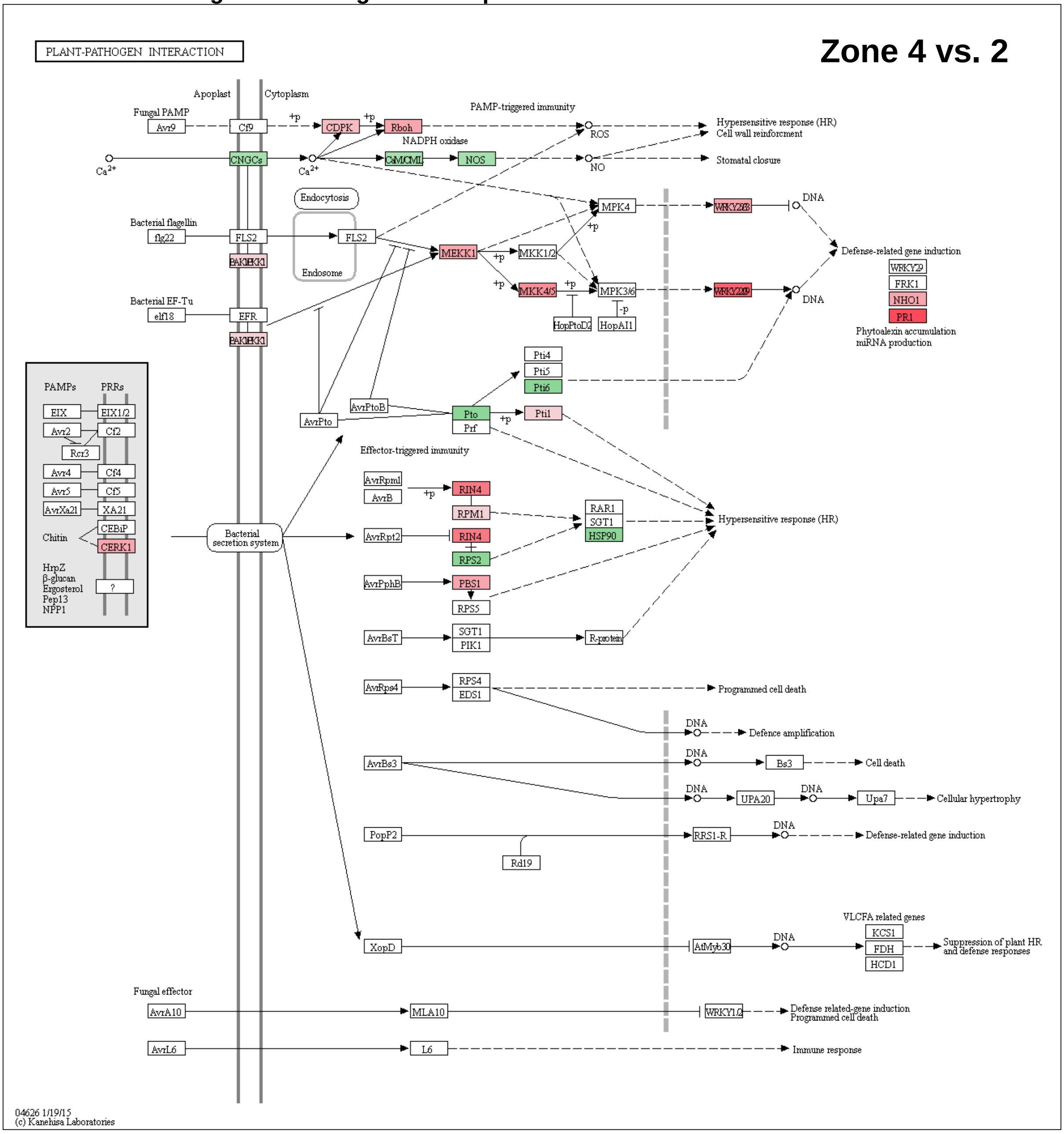

KAAS Kegg mapping of *L. sativa* cv. Tizian DEG's

Only DEG's with p-adjusted values of 0.05 or smaller and absolute fold changes of 2 or higher are depicted

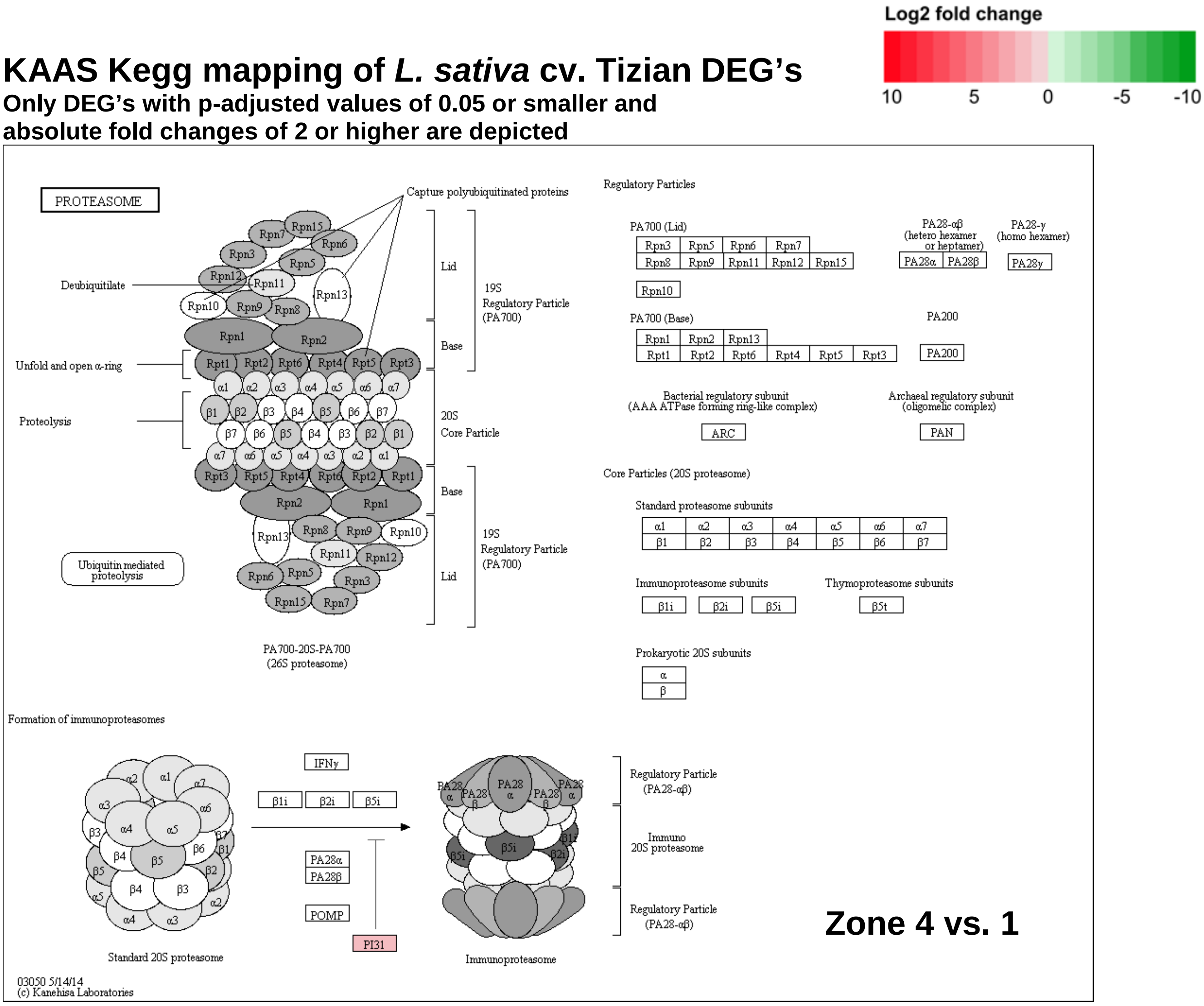

Zone 4 vs. 1

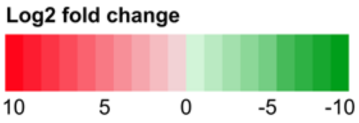

KAAS Kegg mapping of *L. sativa* cv. Tizian DEG’s

Only DEG’s with p-adjusted values of 0.05 or smaller and absolute fold changes of 2 or higher are depicted

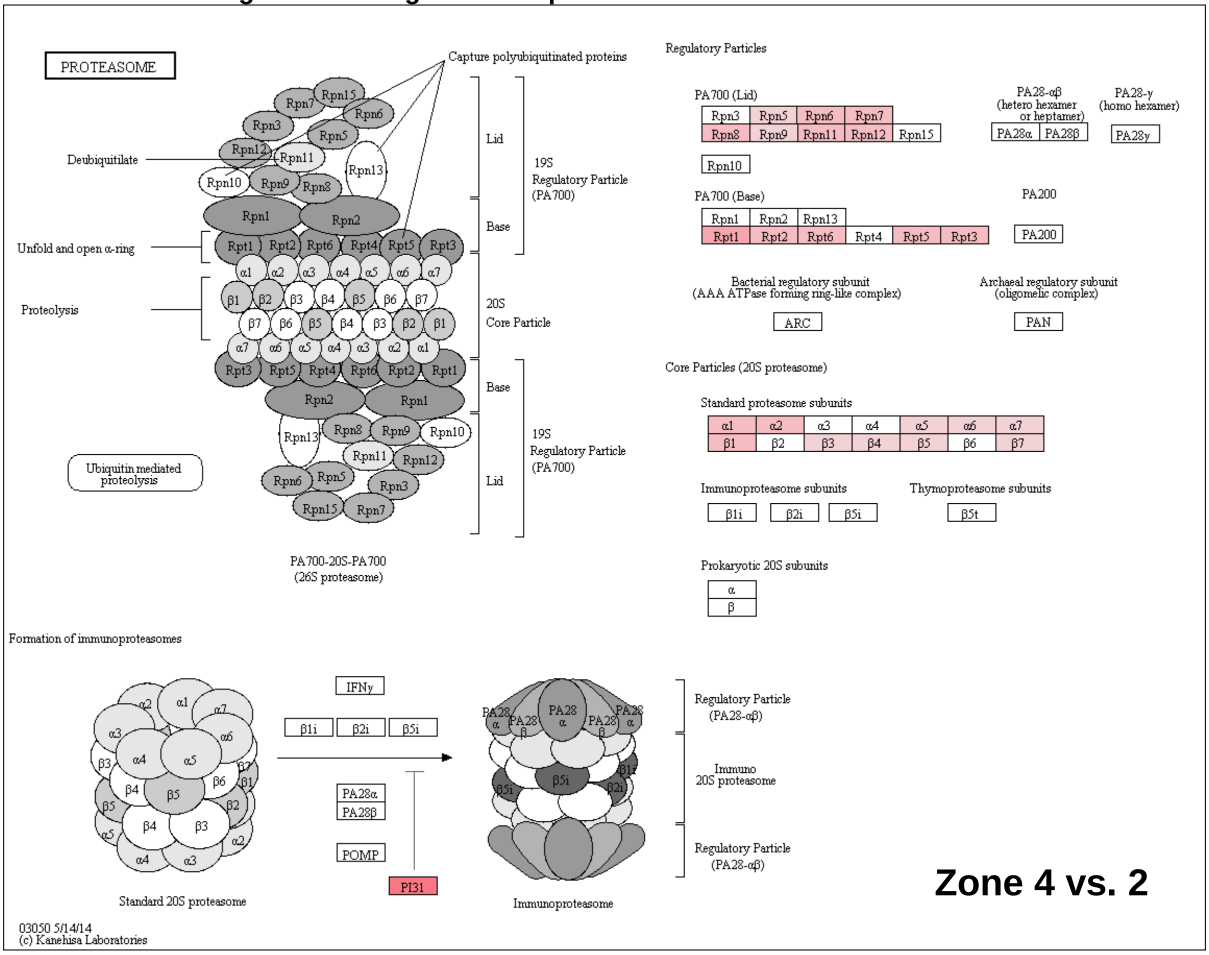

Supplementary Figure S3: KEGG Transcriptome mappings of DEGs based on fold changes. Only DEGs with log2 fold changes of 1 or more and p-adj values of 0.05 or less are depicted. KEGG maps were obtained from www.kegg.jp[40-43]
